# Exploring the Nutritional, Rheological, and Textural Properties of Pumpkin Seed Flour‐Enriched Biscuits in Relation to Their Storage Stability

**DOI:** 10.1002/fsn3.71089

**Published:** 2025-10-24

**Authors:** Muhammad Tayyab Arshad, Nosiba S. Basher, Ali Ikram, Muhammad Ahmad, Nasir A. Ibrahim, Ammar Al‐Farga, Kodjo Théodore Gnedeka

**Affiliations:** ^1^ University Institute of Food Science and Technology The University of Lahore Lahore Pakistan; ^2^ Functional Food and Nutrition Program Center of Excellence in Functional Foods and Gastronomy, Faculty of Agro‐Industry, Prince of Songkla University Songkhla Thailand; ^3^ Department of Biology, College of Sciences Imam Mohammad Ibn Saud Islamic University (IMSIU) Riyadh Saudi Arabia; ^4^ Department of Food Science and Technology Nur International University Lahore Pakistan; ^5^ Biochemistry in Department of Biological Sciences, College of Science University of Jeddah Jeddah Saudi Arabia; ^6^ Togo Laboratory: Applied Agricultural Economics Research Team (ERE2A) University of Lomé Lome Togo

**Keywords:** fiber, gluten, protein, pumpkin

## Abstract

This study evaluated the nutritional and rheological properties of biscuits formulated with wheat flour (WF) and pumpkin seed flour (PSF) blends in different ratios (100:0, 95:5, 90:10, 85:15, and 80:20). Pumpkin seeds were ground and analyzed for moisture (3.93%), ash (0.64%), fat (23.74%), fiber (30.45%), protein (19.83%), and nitrogen‐free extract (21.39%). Composite dough rheology was assessed using farinograph, mixograph tests, and near‐infrared (NIR) analysis. Biscuits were stored for 60 days at a relative humidity (50% ± 5%) and temperature (25°C ± 2°C) and evaluated every 15 days interval for proximate, textural (20 mm diameter cylindrical probe), color, physical, and sensory properties. The farinographic analyses showed that the highest water absorption (63.17 mL/100 g) and dough development time (5.14 min) were observed with 20% PSF, although dough stability decreased (3.14 min). The addition of PSF to biscuits increased their nutritional and textural properties. The D3 (85:15) formulation exhibited optimal texture, color, and nutritional quality. During storage, D3 retained moisture (4.92%), ash (1.45%), fiber (7.86%–7.91%), and texture (hardness: 59.62–59.69 N), achieving the highest overall acceptability score (8.33).

## Graphical abstract

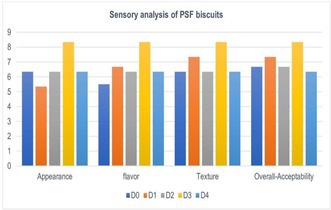

## 1 Introduction

Pumpkin ( *Cucurbita pepo* ), an annual plant belonging to the Cucurbitaceae family, is monoecious. Although pumpkin is primarily considered a vegetable, it is also technically considered a fruit. *Cucurbita pepo*, *C. mixta*, *C. maxima*, and *C. moschata* are common names of squash species that resemble gourds and belong to the Cucurbitaceae family (Aziz et al. 2023). Although pumpkins have various shapes, they are frequently oval or oblong. The smooth rind varied in color from variety to variety. Some fruits are orange‐yellow, white, crimson, gray, pale, or dark green. Pepitas or pumpkin seeds are flat, dark green, and covered with a yellow‐white husk. Pumpkins are farmed worldwide for many reasons, including commercial and decorative sales and agricultural use (Sharma et al. 2020). With an annual production of 8,427,676 tons, China is the world's largest producer outside its borders. Pakistan produces 267,563 tons of pumpkin per year (Hussain et al. 2021).

Pumpkin is a functional food used in several industries such as dairy, meat, drinks, and baking. Additionally, adding pumpkin flour to dough may strengthen the gluten network, promote bread rise, and maintain gas cell structure. By interacting with gluten proteins through polysaccharides and pectic compounds, pumpkin flour or puree improves gluten activity by boosting the viscosity, gas retention, and network strength. Bayramov et al. (2022) reported that pumpkin puree (5%–25%) increased raw gluten strength from 68.5 to 94.7 units, indicating increased resilience and mechanical stability, even without changing the amount of gluten. However, these elements contribute to enhanced nutritional and functional aspects (gluten network enhancement and gas cell structure maintenance) (Melese and Keyata 2023). Valuable functional nutrients are abundant in pumpkin seeds (Singh and Kumar 2022).

Pumpkin seeds are rich in calcium (Ca), magnesium (Mg), potassium (K), phosphorus (P), and manganese (Mn) and are comparatively low in sodium (Na). In addition, trace metals, including zinc (Zn) and iron (Fe), can be found in pumpkin seeds (Hussain et al. 2023). Pumpkin has drawn scientific attention owing to its extensive use in traditional medicine for many ailments, including antidiabetic, antihypertensive, anticancer, immunomodulatory, antibacterial, anti hypercholesterolemic, intestinal antiparasitic, anti‐inflammatory, and analgesic effects (Wal et al. 2024; Batool et al. 2022). Polysaccharides, fatty acids, amino acids, phenolics, carotenoids, flavonoids, minerals, and volatile compounds are abundant in pumpkin (Hagos et al. 2023; Hagos et al. 2022). A recent examination of pumpkin powder production, characterization, food application, and biological studies highlights its potential as a composite flour technology for fortifying white flour in biscuit production (Hussain et al. 2023).

Biscuits that include a mixture of pumpkin powder have been found to have increased nutritional value and improved texture and sensory attributes (Malkanthi and Umadevi 2018). Baked goods can be produced with process efficiency and product quality that meet the specified criteria by incorporating pumpkin component powders into composite flours (Ikram et al. 2024; Baqoyeva et al. 2023). Whole WF can be mixed with germinated PSF to boost the nutritional fiber, mineral content, and protein quality of biscuits. Pumpkin flour can be incorporated into food using composite flour technology, which is ideal for creating baked goods such as cookies and bread (Ikram et al. 2021). Baked products made from cereal flour are well liked globally and offer an excellent opportunity to incorporate nutritional elements from various types of flours (Suleman et al. 2023; Saeed et al. 2021). There are various challenges in the development of biscuits enhanced with PSF. Existing published research frequently concentrates on basic nutritional improvements without thoroughly addressing customer preferences or sensory appeals. Rheological changes that occur during processing and their effects on large‐scale production have not received much attention. Furthermore, there is a lack of thorough information regarding the long‐term storage stability of these products under various environmental conditions, which presents difficulties for their commercial viability. Despite its well‐known nutritional benefits, the impact of PSF on the rheological, textural, and storage stability properties of baked goods has received little attention. Limited research has examined how its inclusion affects biscuit quality over shelf life, leaving a gap in understanding its functional role in the development of products. This study examined the combined nutritional, rheological, textural, and storage stability impacts of PSF in biscuits. It fills the gap between the nutritional benefits and functional performance of baked goods. The main objective of this study was to explore the nutritional profile, dough behavior, and physical properties, including the textural, color, and sensorial profile of PSF dough biscuits.

## 2 Material and Methods

### 2.1 Procurement of Raw Materials

Pumpkin seeds (2 kg) were purchased from a local market in Lahore, Pakistan, and subsequently placed in a sealed ziplock bag to prevent additional contamination. Subsequent examinations were performed at the Food Analysis Lab, University Institute of Food Science and Technology, University of Lahore between September 2023 and January 2024.

### 2.2 Sample Preparation

The pumpkin seeds were sun‐dried under natural sunlight for approximately 8 h per day for four consecutive days, with ambient temperatures ranging from 35°C to 45°C. The seeds were dried until they reached a moisture content of approximately 10%–12%, as measured using a moisture meter, to ensure suitability for subsequent analysis and screening to eliminate impurities. The seeds were crushed into a fine powder using an electric grinder to produce flour. After packing in polythene zip bags, the flour was stored at room temperature for subsequent analysis.

### 2.3 Proximate Analysis and Dietary Fiber

The moisture, ash, crude fat, crude fiber, crude protein, and nitrogen‐free extract (NFE) content of PSF were analyzed using the American Association of Cereal Chemists. Approved Methods Committee (2000) method. PSF was analyzed for soluble, insoluble, and total dietary fiber content using the Megazyme assay kit, as described by the protocol cited in method no. 32‐07 American Association of Cereal Chemists. Approved Methods Committee (2000). Heat‐stable α‐amylase was used to disperse the flour in a buffer solution and incubate it for 40 min at 95°C–100°C (Wang et al. 2022).

$$\begin{array}{c} \mathrm{NFE}\;\text{Content}=\\ 100-\left(\text{crude protein}\%+\text{crude fiber}\%+\text{crude}\;\mathrm{fat}\%+\mathrm{ash}\%+\text{moisture}\%\right) \end{array}$$

### 2.4 Mineral Analysis

Digestion primarily serves to eliminate organic biomass in the sample, facilitating a clear evaluation of inorganic heavy metal levels. In this study, wet digestion was performed. Specifically, 5 g of the material was transferred to a conical flask for digestion, and then 10 mL of concentrated nitric acid (HNO_3_) and 5 mL of sulfuric acid (H_2_SO_4_) were added. A blank sample was also prepared in a digestion conical flask without the sample by adding 10 and 5 mL of nitric acid and sulfuric acid, respectively (Suleman et al. 2023). The mineral composition of PSF was analyzed according to the AOAC (2012) guidelines, following wet digestion in a di‐acid mixture of HCLO_4_: HNO_3_ in a 3:7 ratio. To measure potassium (K) and sodium (Na) using a Flame Photometer −410 (Sherwood Scientific Ltd.). Whereas calcium (Ca), magnesium (Mg), phosphorus (P), iron (Fe), manganese (Mn), and zinc (Zn) were measured using an Atomic Absorption Spectrophotometer.

### 2.5 Swelling, Water Holding and Water Retention Capacity

The swelling capacity of PSF was analyzed using a modified method described by Rosell et al. (2009). The sample was poured into a 100 mL graduated cylinder until it reached the 10‐mL level. 50 mL was the total capacity after the addition of the distilled water. The graduated cylinder was inverted and its top was securely covered and mixed. After 2 min, the suspension was inverted once more and allowed to stand for a further 8 min. After the eighth minute, the sample's volume was measured. The water‐holding capacity of flour samples was ascertained (American Association of Cereal Chemists (AACC), 1990). A vortex mixer (Labnet, VX‐200, USA) was used to combine 5 g of the weighed flour with 25 mL of water for 10 s for each measurement. Each sample was then centrifuged for 15 min at 1000 × *g* using a Kubota General‐Purpose model 4000 in Japan. After discarding the supernatant, the water‐holding capacity was determined using the formula (Sangnark and Noomhorm 2003) and the modified Chantaro et al. (2008) method was used to calculate the water retention capacity.

### 2.6 Antioxidant Properties

#### 2.6.1 Determination of Total Phenolic Compounds

Total phenolic compounds (TPC) are the most popular assays for determining the antioxidant capacity of tested substances. Equal amounts of sample and FC reagent were mixed with 500 μL of distilled water and kept accessible for 5 min, after which 4.5 mL of 7% Na_2_CO_3_ was added and kept accessible for 90 min. Absorbance was measured at 760 nm using a spectrophotometer (IRMECO, U2020). Following this procedure, the total phenolic content was assessed in terms of gallic acid equivalents in aqueous solvents (mg gallic acid/g) (Sengul et al. 2009).

#### 2.6.2 1,1‐Diphenyl‐2‐Picrylhydrazyl Radical Scavenging Assay

The most widely used test for determining antioxidant capability is the DPPH (1,1‐diphenyl‐2‐picrylhydrazyl) free radical scavenging ability test. Briefly, the sample and a 0.12 mM DPPH solution were combined in a test tube at a ratio of 4:1 (sample to DPPH solution) and then left in the dark for 30 min. Subsequently, the absorbance at 520 nm was measured using a UV/Visible Spectrophotometer and a control or blank sample (Tomsone et al. 2012).

### 2.7 Preparation of Composite Dough

The wheat and pumpkin seed flours were mixed at different ratios to prepare the dough. In the composition, D_o_ is a control group and consists of only WF (100%); D_1_ consists of 95% WF and 5% PSF; D_2_ consists of 90% WF and 10% PSF; D_3_ comprises 85% WF and 15% PSF; D_4_ consists of 80% WF and 20% PSF. The treatment plans are presented in Table 1.

**TABLE 1:** Treatment plan.

Dough	WF (%)	PSF (%)
D0	100	0
D1	95	5
D2	90	10
D3	85	15
D4	80	20

*Note:* D0 is a control group and consists of only WF (100%). D1 is the first treatment group and consists of 95% WF and 5% PSF. D2 is the second treatment group and consists of 90% WF and 10% PSF. D3 is the third treatment group comprising 85% WF and 15% PSF. D4 is the fourth treatment group and consists of 80% WF and 20% PSF.

Abbreviations: D0, control sample; PSF, pumpkin seed flour; WF, wheat flour.

### 2.8 Rheological Analysis of Composite Flour

#### 2.8.1 Farinographic Study

The AACC (2000) technique was followed using a farinograph (Brabender D‐4100 SEW, Germany) for the test. The physical dough qualities of composite flour (WF and PSF) blends at various concentrations were assessed. As detailed below, the farinograph was analyzed for several features, such as water absorption, dough stability, dough development time, mixing tolerance index, and dough softening.

#### 2.8.2 Mixographic Study

The mixing behavior of WF combined with varying amounts of PSF was investigated using a mixograph (National NSI‐33R). The mixture investigation was conducted using the AACC (2000) technique no. 54‐40A. Ten grams of composite flour were added to the mixing bowl in the mixograph. After 1 min of dry mixing, six milliliters of water were added and the mixing process was continued for 10 min. Three replicates of each treatment were used to obtain the mean value after the test was conducted thrice.

#### 2.8.3 NIR Analysis

Near‐infrared (NIR) analysis was used to determine the moisture, protein, water absorption, ash, and gluten content (wet, strong, weak, and dry). Gluten from different sample treatments (D0, D1, D2, D3, and D4) was washed using an automatic gluten‐washing apparatus to remove starch, pentosans, and water‐soluble proteins. The wet gluten was centrifuged, and the gluten index (GI) was calculated by measuring the ratio of gluten that passed through and remained on a sieve. Wet gluten was dried, and the weight difference between wet and dry gluten was used to measure the water‐binding capacity (WBC). Finally, the quantities of wet and dry gluten were expressed as percentages of the original sample weight. NIR spectroscopy can be used to predict the calorific value, crude ash, and moisture content of a single biomass source (Fagan et al. 2011).

### 2.9 Product Development and Storage Stability

The method of Kaur et al. (2015) was used with some adjustments to prepare the biscuits (Table 2). Biscuits were prepared from composite flour in the treatment plan, as mentioned above. Biscuits were baked in an oven (190°C, 12 min). The baked biscuits were stored at room temperature in airtight polythene bags for further analysis. The prepared biscuits with different treatments (D0, D1, D2, D3, and D4) were stored for 60 days at a relative humidity of 50% ± 5% and a temperature of 25°C ± 2°C, and biscuits were packaged in airtight, food‐grade polyethylene bags to prevent moisture ingress and contamination. The analysis was performed every 15 days. The 60‐day storage stability was chosen to represent the normal commercial shelf life of biscuits, allowing the evaluation of potential changes under realistic storage conditions. This period allows for a realistic comparison between PSF‐based and normal biscuits in terms of stability and customer acceptability.

**TABLE 2:** Recipe of biscuits (Kaur et al. 2015).

Ingredients	Quantity
Pumpkin flour	As mentioned in the treatment plan
Wheat flour	As mentioned in the treatment plan
Sugar	100 g
Milk (fresh)	100 mL
Whole egg	1/(50–55 g)
Baking powder	2 g
Salt	½ tsp
Cooking oil	150 mL
Vanilla essence	2 drops
Total weight of biscuit	5 g

### 2.10 Proximate Analysis of PSF Biscuits

The proximate composition of the biscuits was examined as a part of the centesimal composition using the AACC 2000 method.

### 2.11 Texture Analysis of PSF Biscuits

Biscuit texture was analyzed using a Texture Analyzer (TA‐XT2, Plus, Stable Microsystems, Surrey, UK) connected to a computer, following the method outlined by Piga et al. (2005), with some modifications. Version 4.0.9.0 of the Texture Expert program was used to analyze the data. A cylindrical probe with a 20 mm diameter was used to perform measurements in a cup that was 7 cm in diameter and 3.5 cm in height. It was moved at a speed of 100 mm/s until it reached a final penetration depth of 12 mm. The probe had a 10 N sensor attached to it. Each formulation was measured three times, and the mean values were calculated.

### 2.12 Color Evaluation of PSF Biscuits

The color of the biscuits was estimated using a CIE‐Lab Color Meter (CIELAB SPACE, Color Tech‐PCM, USA). For the experiment, 5 g of each respective biscuit was taken, and color values like *L (lightness and darkness), *a*٭ (green to red chromaticity), *b*٭ (blue to yellow chromaticity), c٭ (color saturation), *h*٭ (hue angle) were recorded.

### 2.13 Physical Properties of PSF Biscuits

The AACC 2000 procedure was used to measure the width, thickness, and spread ratio of biscuits. The thickness, weight, diameter, and spread ratio of the biscuits were measured. A computerized weighing balance was used to determine the weights of the biscuits. The thickness and diameter of the biscuits were determined using the Vernier caliper, where *W* is the biscuit diameter and *T* is its thickness. The spread ratio was computed by dividing the two variables using the formula (*W*/*T*). Three replicates were performed for each measurement.

### 2.14 Sensory Evaluation of PSF Biscuits

The nine‐point Hedonic Scale System assessed the sensory quality of biscuits on day 1 only using attributes such as appearance, flavor, texture, and overall acceptability, where nine were extremely like and one was extremely dislike, and panelists (consisting of 30 men and 20 women in the age range of 25–35) were asked to assess the sample. A voluntary panel of 50 judges from the Department of Food Science and Technology of the University of Lahore made up a panel for sensory analysis (Meilgaard et al. 2007). All judges were trained, and they were asked to wash their mouths for accurate and better results. All samples were coded to facilitate the judges.

### 2.15 Statistical Analysis

Data were entered, managed, and analyzed using Statistix 8.1. Two‐way ANOVA was applied according to the data. LSD was applied to all storage treatment results. Means ± SDs were calculated for continuous data. Statistical significance was set at *p* ≤ 0.05. The significance level was set at *p* < 0.05, based on the standard criteria for rejecting the null hypothesis. Results are presented as the mean ± standard deviation from triplicate analyses. Where appropriate, 95% confidence intervals were calculated to assess the magnitude and precision of observed effects. All analyses were performed in triplicates.

## 3 Results and Discussion

### 3.1 Proximate Analysis of PSF

**FIGURE 1:**
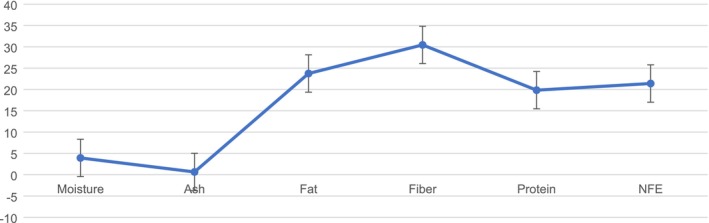
Proximate analysis of PSF.

A proximate analysis of the PSF is shown in Figure 1. This study found that PSF had high fat (23.74%) and protein content (19.83%). The fiber and moisture contents in the PSF were 30.45% and 3.93%, respectively. Conversely, the NFE content was 21.39% and the ash content was 0.64%. In their 2012 study, Elinge et al. examined the physicochemical characteristics, antioxidant potential, and use of fiber from pumpkin ( *Cucurbita pepo* L.) seeds and rinds as a component in baked goods. The protein, crude fiber, crude fat, crude protein, and ash contents were 4.32%, 31.48%, 24.27%, 20.21%, and 0.68%, respectively. These results are consistent with those of the current study. Similar results were reported by Moraes et al. (2024). Another study concluded that the proximate analysis of PSF was within the same range (Adelerin et al. 2024).

### 3.2 Mineral Analysis of PSF

**FIGURE 2:**
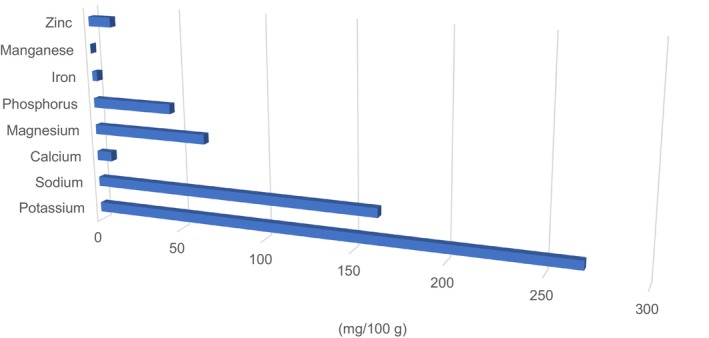
Minerals composition of PSF.

The mineral analysis of the PSF is presented in Figure 2. Based on the results of this study, potassium (269 mg/100 g) was the most abundant mineral in the PSF. The amount of sodium in PSF was 164 mg per 100 g, the calcium concentration was 8.76 mg per 100 g, and the magnesium content in PSF was 66.44 mg/100 g. In contrast, PSF contained 46.67 mg of phosphorus per 100 g and an iron value of 2.97 mg/100 g. Mn was the least abundant among all the minerals analyzed in PSF (0.04 mg/100 g). The zinc content of PSF is comparatively high at 13.13 mg/100 g. These results are comparable to those of Elinge et al. (2012), who reported that pumpkin seeds are a good source of minerals, particularly K, Ca, Na, Mg, P, Zn, and Fe. Similar results were reported by Yetesha et al. (2023). Ayo et al. (2024) also found that the mineral content of PSF was in the same range.

### 3.3 Dietary Fiber Contents of PSF

**FIGURE 3:**
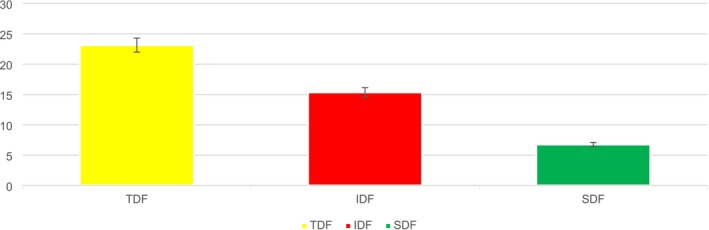
Dietary fiber contents of PSF.

The total (TDF), insoluble (IDF), and soluble (SDF) dietary fiber contents in PSF are presented in Figure 3, with 23.14%, 15.36%, and 6.77%, respectively. In a study by Alshehry (2020), the functional properties, phytochemicals, and vitamins of pumpkin seed powders were assessed by replacing 72% of WF with cookies. The results indicated that the percentages of total, insoluble, and soluble dietary fiber in pumpkin seeds were 24.15%, 16.37%, and 7.78%, respectively. These results are similar to those of the present study. Aktaş and Gerçekaslan (2024) explored the same findings.

### 3.4 Swelling, Water Retention, and Water‐Holding Capacity of PSF

**FIGURE 4:**
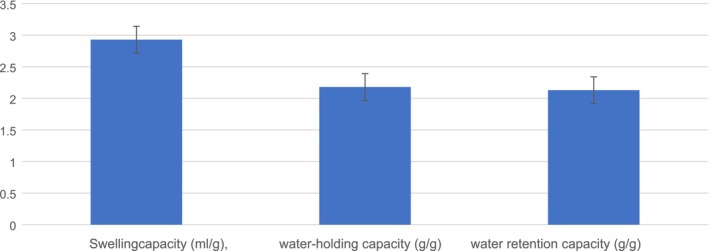
Swelling, water retention, and water‐holding capacity of PSF.

As shown in Figure 4, PSF displayed a minimal swelling capacity of 2.93 mg/g, possibly because of the fat content in the seeds. According to Sowbhagya et al. (2007), the presence of residual oil within the fiber matrix of a sample hinders the ingress of water molecules, resulting in a reduced swelling capacity. Additionally, PSF exhibits a notable water‐holding capacity of 2.18 g/g, which is attributed to its soluble dietary fiber content. The results showed that the water retention capacity of PSF is 2.13 g/g. The present results are in accordance with those of Nyam et al. (2013), who investigated the proximate composition, functional characteristics, and antioxidant activity of pumpkin seeds and rinds. Based on his research, pumpkin seeds exhibit a swelling capacity of 3.25 mg/g, a water‐holding capacity of 2.47 g/g, and a water retention capacity of 2.58 g/g.

### 3.5 Antioxidant Activity of PSF

**FIGURE 5:**
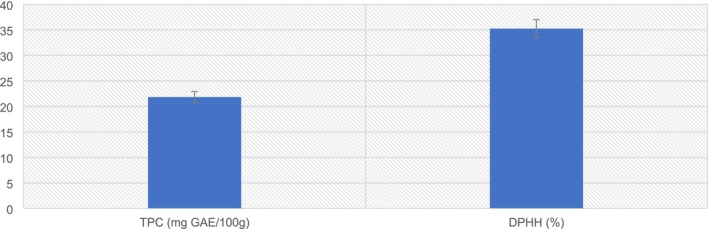
Antioxidant properties of PSF.

PSF showed a DPPH radical scavenging activity of 35.24%. PSF's total phenol content showed 21.83 mg GAE/100 g dry weight (Figure 5). These results are in a similar direction as suggested by Nyam et al. (2013); the pumpkin seeds exhibited a total phenolic compound (TPC) of 22.92 mg GAE/100 g dry weight and a DPPH radical scavenging activity of 36.97%.

### 3.6 Rheological Properties of Composite Flour

#### 3.6.1 Farinograph Analysis

The farinographic and mixographic properties of the PSF biscuits are presented in Table 3.

**TABLE 3:** Mean values of rheological analysis of composite flour.

Sample	Farinograph				Mixograph	
	Water absorption (%)	Dough development time (min)	Dough stability (min)	Mixing tolerance index (BU)	Mixing time (min)	Peak height (BU)
D0	59.70 ± 0.03a	3.93 ± 0.05a	5.15 ± 0.02a	29.27 ± 0.05a	2.56 ± 0.01a	56.32 ± 0.05a
D1	59.92 ± 0.09b	3.98 ± 0.01b	4.30 ± 0.02b	39.72 ± 0.01b	2.38 ± 0.05b	54.54 ± 0.04b
D2	61.30 ± 0.02c	4.13 ± 0.03c	3.78 ± 0.01c	35.82 ± 0.05c	2.21 ± 0.02c	53.20 ± 0.03c
D3	62.67 ± 0.05d	4.92 ± 0.09d	3.32 ± 0.01d	27.43 ± 0.04d	2.18 ± 0.01 cd	52.32 ± 0.03d
D4	63.17 ± 0.02e	5.14 ± 0.04d	3.14 ± 0.01e	26.14 ± 0.03e	2.15 ± 0.02d	51.14 ± 0.03e
Overall mean	61.35	4.42	3.94	31.68	2.29	53.50

*Note:* Values are mean and SD (*n* = 3). Different letters in the row indicate significant differences (*p* < 0.05) among treatments according to LSD test.

Abbreviations: D0, Control; D1, 5% PSF; D2, 10% PSF; D3, 15% PSF; D4, 20% PSF; PSF, pumpkin seed flour.

##### 3.6.1.1 Water Absorption

It is evident that, compared to other concentrations (D1, 59.92%; D2, 61.30%; D3, 62.67%), D0 showed decreased water absorption (59.70%) and the maximum increase (63.17%) was found in (D4) dough with 20% incorporated PSF. PSF has a high fiber and protein content; it may absorb more water when its concentration increases. Hussain et al. (2023) examined pumpkin peel, flesh, and seeds as dried powders at replacement levels of 5%, 10%, and 15% to make biscuits; the results were similar to our findings. There was a significant increase in water absorption levels of 60.98%, 62.35%, 63.78%, and 62.37%, respectively. Apostol et al. (2020) found a non‐significant decrease in dough water absorption when varying amounts of powdered defatted pumpkin seeds were added to WF. The water absorption values for replacement levels of 5%, 10%, and 15% were 59.5%, 59.1%, and 58.5%, respectively, whereas the control dough made entirely of WF had a value of 60%.

##### 3.6.1.2 Dough Development Time

The dough development time (DDT) of D0 was 3.93 min and increased significantly when the PSF concentration increased. After adding 20% PSF, D4 had the highest DDT, whereas D1, D2, and D3 had DDTs of 3.98, 4.13, and 4.92, respectively. The higher protein and fiber content of PSF may cause increased DDT levels.

According to Hussain et al. (2023), biscuits made with varying percentages of pumpkin ( *Cucurbita maxima* ) flesh, peel, and seed powder modifications (0%, 5%, 10%, and 15%) were examined physically and rheologically. According to the study findings, pumpkin seed powder exhibited a DDT of 4.19 min at 0%, 4.52 min at 5%, 4.82 min at 10%, and 5.17 min at 15%. Our results were consistent with the outcomes of this study.

Davoudi et al. (2020) discovered that adding 15% pumpkin powder to composite flours increased the DDT from 3.9 min (control) to 4.9 min, showing a significant boost. Minarovičová et al. (2017) observed that replacing 7.5% WF with powdered pumpkin ( *Cucurbita moschata* ) led to a noticeable increase in DDT. Specifically, the DDT increased from 2.6 min for the control group to 3.1 min with 7.5% pumpkin flour replacement. The DDT levels for 10% and 5% replacement of pumpkin flour were 2.5 and 3.8 min, respectively.

Kundu et al. (2014) found that a bread‐making process using composite flours combined with fiber‐rich pumpkin powder led to a notable increase in DDT levels. Additionally, they associated the increase in DDT levels in flours with pumpkin powder with a higher fiber content, which caused a delay in dough development.

##### 3.6.1.3 Dough Stability

The dough stability study of biscuits prepared with varying percentages of PSF substitution (D0, 0%; D1, 5%; D2, 10%; D3, 15%; and D4, 20%) indicates that the D0 treatment exhibited the highest dough stability, measuring 5.15 min. Furthermore, as the proportion of PSF increased, dough stability decreased noticeably. Specifically, when D4 replaced PSF, the reduction in dough stability was more significant at 3.14 min compared to D1 (4.30 min), D2 (3.78 min), and D3 (3.32 min).

Apostol et al. (2020) reported non‐significant findings regarding dough stability when varying amounts of powdered defatted pumpkin seeds were added to WFs. The pumpkin powder may have disrupted wheat flour gluten and other protein components, causing a decrease in dough stability. According to Hussain et al. (2023), analysis of farinographic investigations revealed that dough stability severely decreased at the 15% replacement level of pumpkin seed powders (5.17 ± 0.01 min). Our findings and outcomes are comparable.

##### 3.6.1.4 Mixing Tolerance Index

According to the findings, replacing the PSF in D0, D1, and D2 with different quantities (0%, 5%, and 10%) significantly increases the mixing tolerance index values. As the concentration of PSF (15% and 20%) increased, the values of the mixing tolerance index began to decrease significantly in D3 and D4. D1 with 5% PSF had the highest MTI value (39.72 ± 0.01), whereas D4 with 20% PSF showed the lowest MTI value (26.14 ± 0.03) because D1 has more gluten networks and stronger gluten structures as compared to D4.

Hussain et al. (2023) performed rheological and physical analyses of biscuits prepared with varying amounts of pumpkin ( *Cucurbita maxima* ) flesh, peel, and seed powder. According to their findings, adding 5% pumpkin seed powder (40.77 ± 0.18) increased the mixing tolerance index. Adding 10% and 15% pumpkin seed powder (36.82 ± 0.11 and 28.47 ± 0.14) decreased the MTI. The outcomes of this study were consistent with our findings.

According to Costa et al. (2018), the MTI of 30% shelled PSF dough was more significant (56.67 BU) than that of the control dough, which contained 30% (unshelled) PSF dough (23.33 BU) and (100%) WF (30 BU). Flour dough (30%) with shelled pumpkin seeds may have a higher MTI, whereas the 30% flour dough with unshelled pumpkin seeds may have a lower MTI. Apostol et al. (2020) mentioned that the influence of the percentage of partially defatted PSF for the mixtures on the rheological quality of flour is minor.

#### 3.6.2 Mixograph Analysis

##### 3.6.2.1 Mixing Time

The sample contained 100% WF (D0), requiring the longest mixing time (2.56 ± 0.01 min). Additionally, the mixing time decreased significantly as the percentage of PSF increased. When 20% of the PSF was replaced with WF, the D4 sample showed the lowest mixing time (2.15 ± 0.02 min). Comparing composite flour dough to a control dough prepared with 100% WF, it was found that D1, D2, and D3 samples had mixing times of 2.38 ± 0.05, 2.21 ± 0.02, and 2.18 ± 0.01 min, respectively, when PSF was used as a replacement. The decrease in mixing time might be attributed to the reduced gluten content. A reduced gluten concentration means fewer proteins to align and link, allowing the dough to reach its peak development more quickly because gluten proteins (gliadin and glutenin) require time to hydrate and form a viscoelastic network. Similar findings were also noted by Hussain et al. (2021) in their research, who helped elucidate the results of the current study. Furthermore, composite flours containing pumpkin powders might require shorter mixing durations because of their lower gluten content compared with the control flours.

##### 3.6.2.2 Peak Height

The D0 (Control) dough with 100% WF had a maximum peak height of 56.32 ± 0.05 BU. Peak height values decreased significantly, with D4 showing a significant decrease at 20% PSF (51.14 ± 0.03 BU). D1, D2, and D3 with PSF replacement levels of 5%, 10%, and 15% exhibited peak heights of 54.54 ± 0.04, 53.20 ± 0.03, and 52.32 ± 0.03 BU.

Compared to the control dough, the decreased peak height of composite flours may be attributed to decreased swelling rates, decreased overall starch content, and increased protein and fiber contents due to the replacement of pumpkin powders (Jukić et al. 2019). A study by Tufail et al. (2020) showed findings similar to the concept that adding cell walls to cereal brands might decrease peak height.

Hussain et al. (2023) conducted rheological and physical studies of biscuits developed with different replacement levels of pumpkin seed powder (0%, 5%, 10%, and 15%) and analyzed the peak height values of 55.61 ± 0.22, 54.23 ± 0.23, 53.34 ± 0.20, 54.39 ± 0.35 BU. These results are consistent with those of the present study. Another study conducted by Apostol et al. (2020) demonstrated WF blends containing 5%, 10%, and 15% partially defatted PSF are suitable for use in bread and other baked goods. However, in order to enhance bread quality, flour combinations containing 15% partially defatted pumpkin seeds are most suitable.

#### 3.6.3 NIR Analysis

The NIR analyses of composite flour for different treatments, that is, D0, D1, D2, D3, and D4, are shown in Table 4. Treatment D0 had the control PSF, whereas D4 had 20% incorporated PSF. D0 had the highest moisture content (14.30%) compared to D1, D2, and D3 (13.50%, 12.46%, and 11.50%, respectively). D4 had the lowest moisture content (10.36%) when 20% PSF was added. Compared with moisture, the protein content gradually increased with the addition of PSF because PSF is naturally high in protein; therefore, adding it to the flour blend boosts the overall protein content. The results indicated that the minimum protein content was observed in the D0 treatment (12.43%) and the maximum protein content in D4 (17.33%), whereas D1, D2, and D3 were 13.50%, 14.50%, and 15.86%, respectively. In the treatments, the protein content gradually increased with PSF.

**TABLE 4:** NIR analysis.

NIR analysis	D0	D1	D2	D3	D4
Moisture %	14.30 ± 0.2a	13.50 ± 0.2b	12.46 ± 0.25c	11.50 ± 0.2d	24.50 ± 0.2b
Protein %	12.43 ± 0.25e	13.50 ± 0.2d	14.50 ± 0.2c	15.86 ± 0.2b	17.33 ± 0.15a
Water absorption %	56.70 ± 0.1e	61.50 ± 0.2d	68.06 ± 0.15c	73.30 ± 0.2b	78.50 ± 0.2a
Ash %	1.19 ± 0.01e	2.15 ± 0.01d	3.13 ± 0.01b	3.05 ± 0.02c	4.06 ± 0.02a
Wet gluten %	26.70 ± 0.2a	21.63 ± 0.1d	24.60 ± 0.1c	24.57 ± 0.4c	25.73 ± 0.1b
Strong gluten (g)	23.53 ± 0.1a	20.66 ± 0.1b	16.56 ± 0.1c	11.56 ± 0.2e	12.60 ± 0.1d
Weak gluten (g)	2.50 ± 0.2c	0.30 ± 0.2d	7.50 ± 0.1b	12.60 ± 0.1a	12.70 ± 0.1a
Total weight of gluten (g)	25.66 ± 0.1a	20.57 ± 0.4c	23.66 ± 0.1b	23.76 ± 0.05b	25.60 ± 0.1a
Index (%)	93.00 ± 2b	99.33 ± 1.5a	68.57 ± 0.4c	47.45 ± 0.02e	49.53 ± 0.1d
Dry gluten (%)	8.57 ± 0.4bc	8.33 ± 1.5c	9.63 ± 0.1abc	9.80 ± 0.1ab	10.76 ± 0.1a

*Note:* Values are mean and SD (*n* = 3). Different letters in the row indicate significant differences (*P* < 0.05) among treatments according to LSD test.

Abbreviations: D0, 0% PSF; D1, 5% PSF; D2, 10% PSF; D3, 15% PSF; D4, 20% PSF; PSF, pumpkin seed flour.

The ash contents of D0, D1, D2, D3, and D4 were 56.70%, 61.50%, 68.06%, 73.30%, and 78.50%, respectively. The minimum ash content was observed on D0 (1.19%) and the maximum on D4 (4.06%), whereas D1, D2, and D3 were 2.15%, 3.13%, and 3.05%, respectively. As the PSF incorporation increased, the ash content gradually increased because of the higher mineral content in PSF compared to WF, which resulted in a higher ash content.

The wet gluten ranged from (D1) 21.63% to 25.73% (D4). The wet gluten content of the control group (D0) was 26.70%. The results indicated that the minimum value (21.63%) was observed on D1, whereas the maximum value (26.70%) was observed on D0 because the amount of PSF increased and WF decreased. The wet gluten in the different treatments i.e., D1, D2, D3, and D4 is 21.63 ± 0.1, 24.60 ± 0.1, 24.57 ± 0.4, 25.73 ± 0.1 respectively.

The present study showed strong gluten ranged from (D1) 20.66 g to 12.60 g (D4). Strong gluten levels in the control (D0) were 23.53 g. The results indicated that the minimum value (11.56 g) was observed for D3, whereas the maximum value (23.53 g) was observed for D0. The concentrations of strong gluten in the different treatment groups (D1, D2, D3, and D4) were 20.66 g, 16.56 g, 11.56 g, and 12.60 g, respectively.

The outcomes of this study indicated that the weak gluten ranged from (D1) 0.30 to 12.70 g (D4). The weak gluten content in the control (D0) was 2.50 g. The results indicate that the minimum value (0.30 g) was observed in D1, whereas the maximum value (12.70 g) was observed in D4. The weak gluten content in the different treatments (D1, D2, D3, and D4) was 0.30, 7.50, 12.60, and 12.70 g, respectively.

The findings of this study indicated that the dry gluten content ranged from (D1) 8.33 to 10.76% (D4). The dry gluten content of the control (D0) was 8.57%. The results indicate that the minimum value (8.33%) was observed in D1, whereas the maximum value (10.76%) was observed in D4. The dry gluten content in the different treatments (D1, D2, D3, and D4) was 8.33, 9.63, 9.80, and 10.76 g, respectively.

### 3.7 Proximate Analysis of PSF Biscuits

The moisture content in the PSF biscuits ranged from D0 (4.78%–9.55%), D1 (4.97%–9.75%), D2 (4.89%–9.41%), D3 (4.92%–9.43%), and D4 (4.97%–9.86%) during the storage period (1, 15, 30, 45, 60). The maximum moisture content (9.86%) was observed on D4, followed by D0 (9.55%), D1 (9.75%), D2 (9.41%), and D3 (9.43%) on day 60, whereas the minimum was observed on D0 (4.55%), followed by D1 (5.60%), D2 (5.54%), D3 (5.93%), and D4 (5.95%) on day 15. It is clear from the findings that the changes among the five different variants (D0, D1, D2, D3, and D4) were significantly different. The moisture content of the PSF biscuits increased substantially during the storage period because of the incorporation of PSF. Moisture content was examined because water migration can create textural changes; excess moisture might cause softness or microbiological decomposition, while moisture loss can make biscuits too hard, both of which influence customer approval.

**FIGURE 6:**
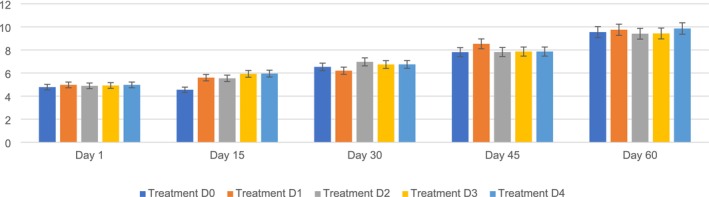
Moisture of PSF biscuits: D0, Control; D1, 5% PSF; D2, 10% PSF; D3, 15% PSF; D4, 20% PSF; PSF, pumpkin seed flour.

These results are similar to those suggested by Kumari et al. 2020, who found that the moisture content in biscuits increased during the storage period. Similar findings regarding moisture content were reported by Bhasker (2016), who observed that the moisture content in biscuits tended to increase during storage. Moisture level is vital for assessing the duration of storage of biscuits. Research on different types of biscuits stored in various packaging materials indicates that the moisture level tends to increase slightly over time owing to the long storage period and increased humidity in the air, affecting the quality characteristics of the biscuits (Adeola et al. 2020; Senarathna and Navaratne 2017). Figure 6 shows the moisture of PSF biscuits.

The crude protein content in the PSF biscuits ranged from D0 (12.55%–12.52%), D1 (27.55%–28.18%), D2 (28.83%–28.53%), D3 (32.89%–32.47%), and D4 (32.53%–32.67%) during storage (1, 15, 30, 45, and 60) periods, respectively. The highest protein content (32.92%) was observed on D3, followed by D0 (12.57%), D1 (27.55%), D2 (28.82%), and D4 (32.57%) on day 45, whereas the lowest was observed on D0 (12.52%), followed by D1 (28.18%), D2 (28.53%), D3 (32.47%), and D4 (32.67%) on day 60. It is clear from the findings that the changes among the five different variants (D0, D1, D2, D3, and D4) were significantly different. The protein content of PSF‐enriched biscuits increases considerably after storage. This increase can be ascribed to moisture loss during storage, resulting in a higher concentration of dry matter, including proteins. The addition of PSF also led to a higher baseline protein level than that of the control. Protein content was tested to assess nutritional stability, as protein breakdown and reactions such as Maillard browning can affect both nutrient value and color.

These results were similar to those of Shukla et al. (2016), who reported that the protein content in biscuits may show different patterns during storage depending on the type of fortification or substitution applied. Adding legumes, such as green gram flour, to WF biscuits can increase protein content and improve their nutritional value.

**FIGURE 7:**
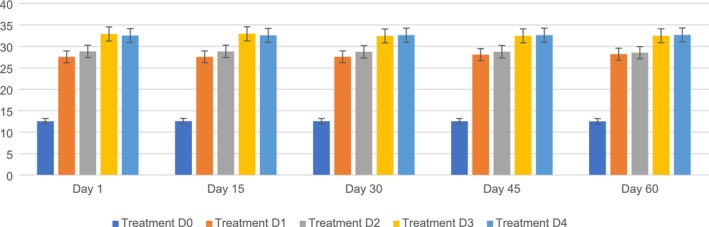
Crude protein of PSF biscuits. D0, control; D1, 5% PSF; D2, 10% PSF; D3, 15% PSF; D4, 20% PSF; PSF, pumpkin seed flour.

Another study by Mohamed et al. (2014) indicated that adding fish protein concentrates, such as carp and shark, can improve biscuit protein content. This increased the protein content compared to that of the control samples. Many studies suggest that it is important to recognize that protein levels can decrease during storage under certain conditions. This underscores the importance of tracking nutritional changes over time to maintain product quality and stability (Shukla et al. 2016; Saeed et al. 2020). Figure 7 shows the crude protein of PSF biscuits.

The crude fat content in the PSF biscuits ranged from D0 (15.56%–15.73%) to D1 (24.54%–24.57%), D2 (24.75%–24.04%), D3 (24.16%–24.60%), and D4 (24.63%–24.77%) during the storage period. The maximum fat content (24.79%) was observed in D2, followed by D0 (15.60%), D1 (24.55%), D3 (24.14%), and D4 (24.72%) on day 15, whereas the lowest fat content was observed on D0 (15.56%), followed by D1 (24.54%), D2 (24.75%), D3 (24.16%), and D4 (24.63%) on day 1. It is clear from the findings that the changes among the five different variants (D0, D1, D2, D3, and D4) were significantly different. The fat content of the biscuits changed throughout storage, with significant increases and decreases observed in all five variations (D0, D1, D2, D3, and D4). These changes can be attributed to various processes, including lipid migration within the biscuit matrix and moisture loss, which result in a relative increase in fat percentage and lipid oxidation over time. The incorporation of PSF, which is high in fat, leads to a greater initial fat content in enriched variations (D1–D4). The observed variances across samples during storage could possibly represent differences in the oxidative stability of lipids and experimental variability in fat analysis.

These results are in line with the findings of Kumari et al. (2021), who mentioned that including PSF in biscuits has been observed to have a notable effect on fat levels during storage. The high oil content in pumpkin seeds directly contributes to the overall fat content of the biscuits. According to a previous study, substituting white flour with PSF resulted in an increase in fat content in biscuits, particularly when the replacement level reached 15%. Analysis of the chemical composition of orange seed flours revealed varying levels of fat content, which could affect the fat content of the end product when used in biscuits. Moreover, nutritional assessment of pumpkin seed meal biscuits indicated a fat content of 18.0%, suggesting that biscuits containing PSF had a higher fat content. Hence, incorporating PSF in biscuit production may increase fat content, which could further develop during storage periods (Hussain et al. 2023; Suleman et al. 2023; Gao et al. 2022).

**FIGURE 8:**
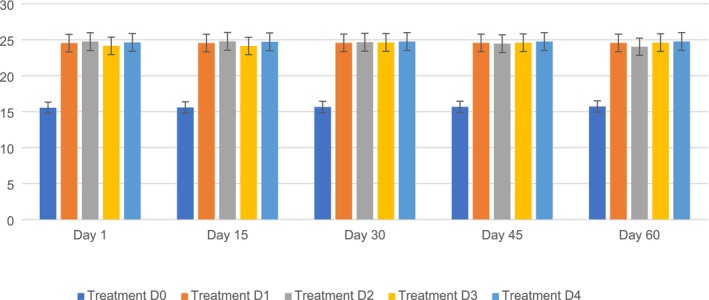
Crude fat of PSF biscuits. D0, Control; D1, 5% PSF; D2, 10% PSF; D3, 15% PSF; D4, 20% PSF; PSF, pumpkin seed flour.

Another study by Hussain et al. (2023) reported that incorporating PSF into biscuit recipes significantly increased the fiber content of the biscuits, making the product healthier. Furthermore, substituting white flour with pumpkin powders, such as PSF, leads to a substantial increase in the fiber content in biscuits, particularly when higher replacement levels are used. Figure 8 shows the crude fat of PSF biscuits.

The crude fiber content in the PSF biscuits ranged from D0 (5.53%–5.63%), D1 (7.51%–7.63%), D2 (7.75%–7.86%), D3 (7.86%–7.91%), and D4 (7.93%–7.92%) during the storage period, respectively. The highest values for the control sample (D0) were measured on day 30 (5.66%), for sample D1 on day 30 (7.57%), for sample D2 on day 60 (7.86%), for sample D3 on days 45 and day 60 (7.91%). In contrast, the lowest fiber content was observed on D0 (5.53%), followed by D1 (24.54%), D2 (24.75%), D3 (24.16%), and D4 (24.63%) on day 1. It is clear from the findings that the changes among the five different variants (D0, D1, D2, D3, and D4) were significantly different. The addition of PSF significantly increased the fiber content of biscuits during storage. Ash and fiber were added to ensure mineral and dietary fiber stability, which contributed to the functional health benefits of the biscuits.

These results agree with those of Shevchenko et al. (2022), who reported that adding PSF to biscuits boosts their fiber content and improves their nutritional value. Pumpkin seeds are high in fiber, and PSF contains 3.5 times more fiber than WF. The heightened fiber content not only enhances the nutritional makeup of biscuits, but also provides potential health benefits, making them a healthier snacking choice.

**FIGURE 9:**
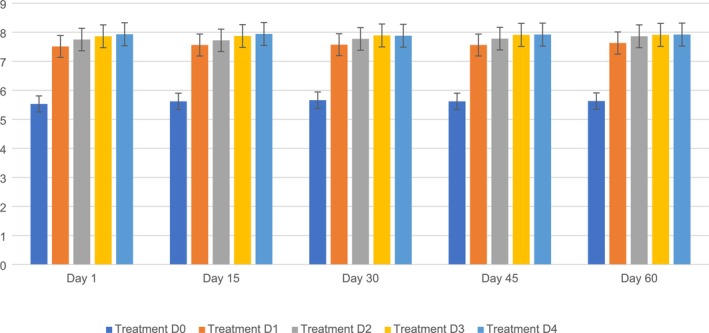
Crude fiber of PSF biscuits. D0, Control; D1, 5% PSF; D2, 10% PSF; D3, 15% PSF; D4, 20% PSF; PSF, pumpkin seed flour.

Another research conducted by Hussain et al. (2023) concluded that incorporating PSF into biscuit recipes significantly increases the fiber content, making the product healthier. Furthermore, substituting white flour with pumpkin powders, such as PSF, dramatically increases the fiber content in biscuits, particularly when higher replacement levels are used. Figure 9 shows the crude fiber of PSF biscuits.

The ash content in the value‐added PSF biscuits ranged from D0 (1.23%–1.31%) to D1 (0.97%–1.32%), D2 (1.53%–1.56%), D3 (1.45%–6.57%), and D4 (6.57%–6.51%) during the storage period. The maximum ash content (6.57%) was observed on D3, followed by D0 (1.31%), D1 (1.32%), D2 (1.56%), and D4 (6.51%) on day 60. On the other hand, the minimum ash content was observed in D1 (0.9%), followed by D0 (1.23%), D2 (1.53%), D3 (1.45%), and D4 (6.54%) at Day 1. It was clear from the findings that the changes among the five variants (D0, D1, D2, D3, and D4) were significantly different. The ash content of the biscuits increased substantially during the storage period because of the incorporation of PSF. Flour transparency was evaluated based on the mineral content. The mineral composition of the flour is closely related to its storage duration. The mineral levels remained constant when the flour was briefly stored. However, prolonged storage increased the ash content of the flour owing to moisture loss, which increased the mineral content.

These results agree with the findings of Das et al. (2021), who reported that biscuits with PSF showed a notable increase in ash content, and a 30% substitution level resulted in ideal values for ash, fat, and fiber content when stored.

**FIGURE 10:**
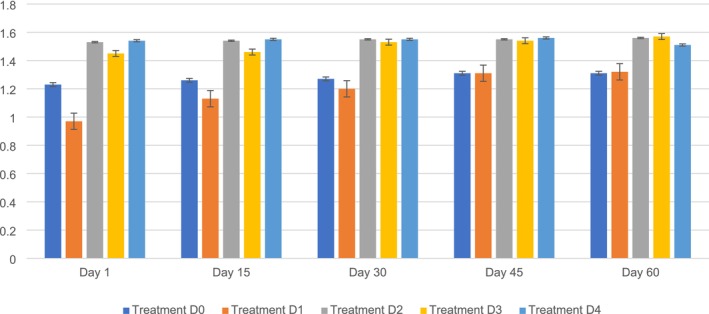
Crude ash content of PSF biscuits. D0, Control; D1, 5% PSF; D2, 10% PSF; D3, 15% PSF; D4, 20% PSF; PSF, pumpkin seed flour.

Another similar study conducted by Gao et al. (2022) concluded that incorporating PSF into biscuits leads to a 1.60% ash content when stored. Several researchers have concluded that incorporating PSF into biscuits causes a noticeable change in the ash content over time. Studies have revealed that adding PSF to biscuits increases ash content, indicating a higher concentration of minerals in the final product. Biscuits with PSF displayed higher ash levels, demonstrating improved mineral profiles with increased amounts of calcium, magnesium, zinc, and iron, compared to regular biscuits. This increase in ash content emphasizes the nutritional advantages of using PSF in biscuit recipes, making it a valuable source of essential minerals to address micronutrient deficiencies in different age groups (Mazaal et al. 2022; Kumari et al. 2021). Figure 10 shows the crude ash content of PSF biscuits.

The present study findings indicated that the NFE content in PSF biscuits ranged from D0 (39.66%–44.75%), D1 (65.56%–71.57%), D2 (67.75%–71.41%), D3 (71.30%–81%), and D4 (76.64%–81.64%) during the storage period. The highest NFE content (81.64%) was noted for D4, followed by D0 (44.75%), D1 (71.57%), D2 (71.41%), and D3 (81%) on day 60. Conversely, the minimum was observed on D0 (39.62%), followed by D1 (66.41%), D2 (68.43%), D3 (72.3%), and D4 (77.77%) on day 15. It is clear from the findings that the changes among the five different variants (D0, D1, D2, D3, and D4) were significantly different. The addition of PSF significantly increased the NFE content of the biscuits during the storage period. The carbohydrate content was assessed for potential alterations caused by starch retrogradation or breakdown, which could affect sweetness, texture, and mouthfeel. Together, these data offer an exhaustive summary of how storage impacts both the quality features and customer acceptability of pumpkin seed‐flour‐based biscuits when compared to normal biscuits.

The present results align with those of Kumari et al. (2020), who reported that using PSF in biscuits can make a big difference in nutritional content, especially the NFE content. PSF contains high levels of protein and fiber, which can improve the nutritional quality of the end product.

**FIGURE 11:**
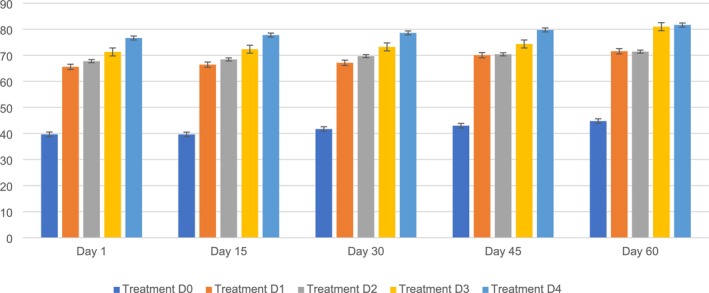
NFE content of PSF biscuits. D0, Control; D1, 5% PSF; D2, 10% PSF; D3, 15% PSF; D4, 20% PSF; PSF, pumpkin seed flour.

According to several studies, incorporating pumpkin seed meal in biscuit recipes elevates dietary fiber levels, resulting in a greater NFE content owing to the carbohydrates in the pumpkin seed meal. Hence, integrating PSF in biscuit manufacturing could be a beneficial approach to increase the NFE content and improve the nutritional value of the final product (Gao et al. 2022; Hussain et al. 2023). The highest percentages of protein (8.8%), ash (1.12%), fiber (4.25%), and total carbohydrates (97.84%) were found in biscuits prepared with a 15% (w/w) PSF ratio (Kumari et al. 2020). Figure 11 shows the NFE content of PSF biscuits.

### 3.8 Texture Analysis

The present study findings indicated the hardness (F) in PSF biscuits ranged from D0 (24.80–28.55N), D1 (56.56–57.47N), D2 (58.56–58.89N), D3 (59.62–59.69N), and D4 (60.52–60.01N) during the storage period, respectively. Hardness increased in biscuits (58.95N) in D2, followed by D0 (27.15N), D1 (56.56N), D3 (59.70N), and D4 (59.69N) on day 45. However, the maximum decrease was noted in D0 (24.80N), followed by D1 (56.56N), D2 (58.56N), D3 (59.62N), and D4 (60.52N) on day 15. The mean hardness (F) values in the PSF‐formulated biscuits were significantly different (*p* ≤ 0.05). The addition of PSF significantly increased the hardness (F) of the biscuits during storage because of the loss of moisture content. Treatment D3 showed significant results regarding the hardness of biscuits incorporated with 15% PSF.

The present outcomes followed those of Khan et al. (2019), who reported that the texture of biscuits can be influenced by the rheological properties of WF when mixed with pumpkin flour. The biscuits were improved using an optimal pumpkin flour concentration of 15. Ingredient selection and proportion significantly affected the hardness of PSF biscuits during storage.

The findings of the present study indicate that the shear and break (F) forces applied on biscuits formulated with PSF in treatments D0 (15.43–13.58N), D1 (2.07–2.14N), D2 (2.23–2.33N), D3 (2.36–2.57N), and D4 (2.66–2.79N) during the storage period. The maximum shear and break forces applied to the biscuits (15.43N) were observed in treatment D0, followed by D1 (2.07N), D2 (2.23N), D3 (2.36N), and D4 (2.66N) on day 60. In contrast, minimum shear and break forces applied on the biscuits were observed in treatments D1 (2.07N), D0 (15.43N), D2 (2.23N), D3 (2.36N), and D4 (2.66N) on day 15. The mean shear and break (F) force values applied to the PSF biscuits were significantly different (*p* ≤ 0.05), as shown in Table 5. The shear and break forces on the biscuits increased substantially owing to the incorporation of PSF. D3 treatment showed significant results regarding shear and break forces applied to biscuits incorporated with 15% PSF during storage.

**TABLE 5:** Texture analysis of PSF biscuits.

Treatment	Storage					
	Day 1	Day 15	Day 30	Day 45	Day 60	Mean
*Hardness*						
D0	24.80 ± 0.60 k	25.83 ± 0.57j	27.30 ± 0.72i	27.15 ± 0.29i	28.55 ± 0.08 h	26.72E
D1	56.56 ± 0.11 g	56.77 ± 0.21 g	56.79 ± 0.09 g	56.56 ± 0.05 g	57.47 ± 0.02f	56.83D
D2	58.56 ± 0.07e	58.69 ± 0.12e	58.80 ± 0.06e	58.95 ± 0.04e	58.89 ± 0.01e	58.78C
D3	59.62 ± 0.12d	59.68 ± 0.02d	59.81cd ± 0.06e	59.70 ± 0.13d	59.69 ± 0.12d	59.70B
D4	60.52 ± 0.02a	60.43 ± 0.02ab	60.59 ± 0.05a	60.25 ± 0.61a‐c	60.01 ± 0.04b‐d	60.36A
Mean	52.01D	52.28C	52.66B	52.52B	52.92A	
*Shear and Break*						
D0	15.43 ± 0.11a	13.10 ± 0.52c	13.48 ± 0.07b	12.88 ± 0.53c	13.58 ± 0.24b	13.69A
D1	2.07 ± 0.04 m	2.13 ± 0.02 lm	2.12 ± 2.12 lm	2.16 ± 0.01 k‐m	2.14 ± 0.03 Lm	2.12E
D2	2.23 ± 0.02j‐m	2.29 ± 0.05i‐m	2.30 ± 0.01i‐m	2.31 ± 0.01 h‐m	2.33 ± 0.01 h‐m	2.29D
D3	2.36 ± 0.02 h‐l	2.42 ± 0.02 g‐k	2.48 ± 0.03f‐j	2.51 ± 0.01e‐i	2.57 ± 0.02d‐h	2.46C
D4	2.66 ± 0.01d‐g	2.68 ± 0.03d‐f	2.73 ± 0.01d‐f	2.76 ± 0.01de	2.79 ± 0.01d	2.72B
Mean	4.95A	4.52C	4.62 bc	4.52C	4.68B	

*Note:* Values are mean and SD (*n* = 3). Different letters in the columns of each parameter indicate significant differences (*p* < 0.05) among treatments according to LSD test.

Abbreviations: D0, Control; D1, 5% PSF; D2, 10% PSF; D3, 15% PSF; D4, 20% PSF; PSF, pumpkin seed flour.

The present outcomes followed the findings of several studies, which concluded that texture characteristics, such as shear force and break force, are significantly affected by the addition of pumpkin flour during biscuit preparation and may continue to change during storage. Additionally, storage conditions can further impact texture attributes, as chemical changes are inversely related to the overall acceptability scores, potentially influencing shear and break force values over time. Research indicates that as the proportion of pumpkin powder in the biscuit formulation increases, hardness and tractability also increase, resulting in higher shear and break force values (Khan et al. 2019; Kulkarni and Joshi 2013; Baltacıoğlu and Ülker 2017).

### 3.9 Color Analysis

The color analysis (*L*, *a*, *b*, *c* and *h* values) of the PSF biscuits is presented in Table 6. The *L*٭ values of PSF biscuits were analyzed in different treatments, i.e., D0 (40.56–40.71), D1 (48.6–48.82), D2 (48.87–48.96), D3 (48.95–49.10), and D4 (49.14–49.19) during the storage period days, respectively. The findings of the present study indicate that the maximum *L*٭ value of biscuits (49.19) was observed on D4, followed by D0 (40.71), D1 (48.82), D2 (48.96), and D3 (49.10) on day 60. In contrast, the minimum *L*٭ value was noted for biscuits on D0 (24.80), followed by those on D1 (56.56), D2 (58.56), D3 (59.62), and D4 (60.52) on day 1. The means of the *L*٭ value in PSF biscuits was significantly different (*p* ≤ 0.05). According to the results, the D3 treatment showed significant values (*L*٭ value) regarding lightness in biscuits due to the incorporation of 15% PSF, followed by D0, D1, D2, and D4, respectively. Similar results were reported by Balaswamy et al. (2022), who mentioned that the presence of carotenoids in PSF might affect the color attributes of biscuits, contributing to the characteristic color of the product. Consequently, the incorporation of PSF changes the texture of biscuits and influences their color profile, highlighting its significance in product development and storage.

**TABLE 6:** Color analysis of PSF biscuits.

(*L*٭)							
Treatment	*Storage*	*Day 1*	*Day 15*	*Day 30*	*Day 45*	*Day 60*	*Mean*
	D0	40.56 ± 0.01e	40.62 ± 0.02d	40.66 ± 0.01d	40.69 ± 0.01d	40.71 ± 0.01d	40.65E
	D1	48.6 ± 0.05c	48.68 ± 0.02c	48.78 ± 0.02b	48.78 ± 0.02b	48.82 ± 0.01b	48.73D
	D2	48.87 ± 0.01b	48.90 ± 0.01b	48.93 ± 0.01b	48.96 ± 0.03b	48.96 ± 0.01b	48.92C
	D3	48.95e ± 0.02b	48.94 ± 0.04b	49.07 ± 0.02a	49.06 ± 0.01a	49.10 ± 0.01a	49.02B
	D4	49.14 ± 0.02a	49.14 ± 0.02a	49.16 ± 0.02a	49.18 ± 0.01a	49.19 ± 0.01a	49.16A
	Mean	47.22D	47.25C	47.32B	47.33B	47.35A	
(*a*٭)							
Treatment	*Storage*	*Day 1*	*Day 15*	*Day 30*	*Day 45*	*Day 60*	*Mean*
	D0	10.59 ± 0.05de	10.64 ± 0.01de	10.68 ± 0.00d	10.72 ± 0.02d	10.73 ± 0.01d	10.67D
	D1	11.89 ± 0.01c	11.91 ± 0.01bc	11.90 ± 0.01bc	11.91 ± 0.01bc	11.93 ± 0.01bc	11.91C
	D2	11.92 ± 0.02c	11.93 ± 0.01c	11.96 ± 0.01bc	11.91 ± 0.01c	11.84 ± 0.12c	11.91C
	D3	11.92 ± 0.01c	11.98 ± 0.01c	12.05 ± 0.01bc	12.08 ± 0.01ab	12.09 ± 0.01ab	12.02B
	D4	12.13 ± 0.01ab	12.15 ± 0.01b	12.16 ± 0.02b	12.18 ± 0.02ab	12.22 ± 0.01a	12.16A
	Mean	11.69C	11.72B	11.75A	11.76A	11.76A	
(*b*٭)							
Treatment	*Storage*	*Day 1*	*Day 15*	*Day 30*	*Day 45*	*Day 60*	*Mean*
	D0	26.70 ± 0.05c	26.78 ± 0.01c	26.79 ± 0.01c	26.80 ± 0.02c	26.91 ± 0.01bc	26.79A
	D1	26.59 ± 0.05 cd	26.60 ± 0.02 cd	26.64 ± 0.02c	26.71 ± 0.01c	26.77 ± 0.01c	26.66A
	D2	26.81 ± 0.01bc	26.85 ± 0.03c	26.87 ± 0.01c	26.91 ± 0.01b	26.88 ± 0.07bc	26.86A
	D3	26.93 ± 0.01b	26.97 ± 0.01b	26.98 ± 0.00b	27.05 ± 0.04a	27.11 ± 0.01a	27.01A
	D4	27.15 ± 0.01a	27.17 ± 0.00a	23.82 ± 8.63b	27.19 ± 0.01c	27.23 ± 0.01c	24.51B
	Mean	26.83A	26.87A	26.22B	25.93B	25.98B	
(*c*٭)							
Treatment	*Storage*	*Day 1*	*Day 15*	*Day 30*	*Day 45*	*Day 60*	*Mean*
	D0	27.59 ± 0.03 l	27.63 ± 0.01kl	27.67 ± 0.01kl	27.67 ± 0.01kl	27.74 ± 0.02 k	27.66E
	D1	28.36 ± 0.42j	28.64 ± 0.02i	28.69 ± 0.01hi	28.77 ± 0.02ghi	28.81 ± 0.01f‐h	28.65D
	D2	28.84 ± 0.03e‐g	28.90 ± 0.01d‐g	28.91 ± 0.01d‐g	28.93 ± 0.01d‐f	28.92 ± 0.01d‐f	28.90C
	D3	28.94 ± 0.01d‐f	28.96 ± 0.02c‐e	28.97 ± 0.01c‐e	28.99 ± 0.01d	29.09 ± 0.01a‐c	28.99B
	D4	29.12 ± 0.02ab	29.16 ± 0.01a	29.14 ± 0.03a	29.19 ± 0.01a	29.22 ± 0.01a	29.17A
	Mean	28.57C	28.66B	28.67B	28.71AB	28.75A	
(*h*٭)							
Treatment	*Storage*	*Day 1*	*Day 15*	*Day 30*	*Day 45*	*Day 60*	*Mean*
	D0	66.57 ± 0.02j	66.62 ± 0.02j	67.37 ± 0.53i	67.68 ± 0.02 h	67.71 ± 0.01d	67.19E
	D1	67.66 ± 0.11 h	67.81 ± 0.06 h	67.83 ± 0.01 g	67.89 ± 0.02 fg	67.91 ± 0.01 cd	67.82D
	D2	67.92 ± 0.4e‐g	67.95 ± 0.01e—	67.96 ± 0.02d	67.97 ± 0.01 cd	67.97 ± 0.02 cd	67.95C
	D3	67.95 ± 0.03e‐g	67.98 ± 0.01bc	68.05 ± 0.05ab	68.08 ± 0.02ab	68.13 ± 0.02ab	68.04B
	D4	68.14 ± 0.02a‐d	68.16 ± 0.01ab	68.16 ± 0.02ab	68.18 ± 0.02a	68.22 ± 0.04a	68.17A
	Mean	67.64C	67.70C	67.87B	67.96A	67.99A	

*Note:* Values are Mean and SD (*n* = 3). Different letters in the columns of each parameter indicate significant differences (*p* < 0.05) among treatments according to LSD test. L* (lightness and darkness); a* (green to red chromaticity); b* (blue to yellow chromaticity); c* (color saturation); h* (hue angle).

Abbreviations: D0, Control; D1, 5% PSF; D2, 10% PSF; D3, 15% PSF; D4, 20% PSF; PSF, pumpkin seed flour.

PSF biscuits showed a* values in different treatments, that is, D0 (10.59–10.73), D1 (11.89–11.93), D2 (11.92–11.84), D3 (11.92–12.09), and D4 (12.13–12.22), during the storage period. The findings of the present study indicated that the maximum a* value of biscuits (12.22) was observed on D4, followed by D0 (10.73), D1 (11.93), D2 (11.84), and D3 (12.09) on day 60. On the other hand, the minimum a* value was noted for biscuits in the treatment D0 (10.59), followed by D1 (11.89), D2 (11.92), D3 (11.92), and D4 (12.13) at Day 1. The mean of a* values in PSF biscuits was significantly different (*p* ≤ 0.05). Based on these results, the D3 treatment, which included 15% PSF, had significant values (*a* value) for biscuit lightness, followed by D0, D1, D2, and D4 in that order. Similar results were reported by Das et al. (2021), who found that adding PSF to biscuits could cause changes in the color a* value over time.

According to several studies, incorporating pumpkin seed powder into biscuit manufacturing improves the visual appearance of the end product. Substituting 5%–10% of the ingredients with pumpkin seed powder resulted in high ratings for color, taste, texture, flavor, and overall approval. Furthermore, the color profile of fresh pumpkins is influenced by carotenoids, with β‐carotene being a key component that gives a distinctive yellow or orange color (Hussain et al. 2023; Balaswamy et al. 2022). Consequently, adding PSF not only boosts the nutritional content of cookies but also affects their color characteristics, resulting in visually appealing treatments that may be more attractive to customers.

The b* values of PSF biscuits were analyzed in different treatments during the storage period: D0 (26.70–26.91), D1 (26.59–26.77), D2 (26.81–26.88), D3 (26.93–27.11), and D4 (27.15–22.23) during the storage period. The findings of the present study indicated that the maximum b* value of biscuits (27.23) was observed on D4, followed by D0 (26.91), D1 (26.77), D2 (26.88), and D3 (27.11) on day 60. In contrast, the minimum b* values were noted for biscuits D1 (26.59), D0 (26.70), D2 (26.81), D3 (26.93), and D4 (27.15) on Day 1. The mean values of b* in the PSF biscuits were significantly different (*p* ≤ 0.05). D3 treatment had the highest lightness (b* value) among the biscuits, followed by D0, D1, D2, and D4.

Similar results depicted according to the findings of Khan et al. (2019), who mentioned that when PSF is added to biscuits, it can cause changes in the color b* value over time. According to several studies, higher levels of seed pumpkin flour resulted in a significant increase in the color values (L*, a*, b*) of the end product, and this pattern persisted during storage at 4°C for 28 days, showing a continued increase. Therefore, adding PSF can influence the color b* value of biscuits, with higher concentrations potentially intensifying the color over time.

The c* values of PSF biscuits were depicted in different treatments, D0 (27.59–27.74), D1 (28.36–28.81), D2 (28.84–28.92), D3 (28.94–29.09), and D4 (29.12–29.22), during the storage period. The findings of the present study indicated that the maximum c* value of biscuits (29.22) was observed on D4, followed by D0 (27.74), D1 (28.81), D2 (28.92), and D3 (29.09) on day 60. In contrast, the minimum c* value was noted for biscuits in treatment D0 (27.63), followed by D1 (28.64), D2 (28.90), D3 (28.96), and D4 (29.16) on day 15. The mean c* values in the PSF biscuits were significantly different (*p* ≤ 0.05).

The D3 treatment showed significant values (c* value) regarding lightness in biscuits because of the incorporation of 15% PSF, followed by D0, D1, D2, and D4. Similar results were reported by Costa et al. (2018), who found that incorporating PSF into biscuits affected the color c* value over time.

Another study on biscuits blended with pumpkin pulp and seed powder showed that adding 5% of this powder led to biscuits that received high ratings for their visual appeal, color, and overall likability, suggesting a favorable influence on the taste and appearance of the product (Jayalath et al. 2024). These results indicate that incorporating PSF can change the color properties of biscuits, potentially improving their visual attractiveness and overall appeal to consumers, thus positioning it as a valuable ingredient for food industry innovation (Malkanthi and Umadevi 2018).

The h* values of PSF biscuits were analyzed in different treatments, that is, D0 (66.57–67.71), D1 (67.66–67.91), D2 (67.92–67.97), D3 (67.95–68.13), and D4 (68.14–68.22), during the storage period. The findings of the present study indicate that the maximum h* value of biscuits (68.22) was observed on D4, followed by D0 (67.71), D1 (67.91), D2 (67.97), and D3 (68.13) on day 60. In contrast, a minimum h* value was noted for biscuits on D0 (66.57), D1 (67.66), D2 (67.92), D3 (67.95), and D4 (68.14) on day 1. The mean h* values in the PSF biscuits were significantly different (*p* ≤ 0.05).

The D3 treatment showed significant values (h* value) for lightness in biscuits because of the incorporation of 15% PSF, followed by D0, D1, D2, and D4. PSF may affect the color h* value of biscuits during storage.

This suggests that a balance between color improvement and overall product appeal was achieved. Consequently, adding PSF to biscuits can improve the color h* value and offer aesthetic appeal and possible health benefits. As pumpkin flour contains carotenoids, it can provide biscuits with a greenish hue, enhancing their visual attractiveness.

### 3.10 Physical Analysis of PSF Biscuits

The physical properties of the PSF biscuits are presented in Table 7. The widths of the PSF biscuits were analyzed in different treatments, that is, D0 (41.24–41.32 mm), D1 (40.80–40.94 mm), D2 (39.97–39.96 mm), D3 (38.98–38.05 mm), and D4 (37.14–37.22 mm) during the storage period. The findings of the present study indicated that the maximum width of PSF biscuits (41.32 mm) was observed in the treatment D0, followed by D1 (40.94 mm), D2 (39.96 mm), D3 (38.05 mm), and D4 (37.22 mm) on day 60. In contrast, the minimum width values were noted for PSF biscuits D4 (37.14 mm), D0 (41.24 mm), D1 (40.80 mm), D2 (39.97 mm), and D3 (38.98 mm) on day 1. The mean widths of the PSF biscuits were significantly different (*p* ≤ 0.05). According to the results, the D4 treatment showed the maximum decrease in width with 20% incorporation of PSF. The width value decreased as PSF incorporation increased. Similar results were reported by Van Toan and Anh (2018), who created biscuits using various amounts of pumpkin flours.

**TABLE 7:** Physical analysis of PSF biscuits.

Treatment	Storage					
	Day 1	Day 15	Day 30	Day 45	Day 60	Mean
*Width (mm)*						
D0	41.24 ± 0.02a	40.92 ± 0.5b	41.27 ± 0.01a	41.25 ± 0.02a	41.32 ± 0.01a	41.20A
D1	40.80 ± 0.01b	40.86 ± 0.01b	40.87 ± 0.01b	40.91 ± 0.02ab	40.94 ± 0.02b	40.87B
D2	39.97 ± 0.01c	39.65 ± 0.5c	39.95 ± 0.01bc	39.95 ± 0.02bc	39.96 ± 0.02bc	39.90C
D3	38.98 ± 0.01c	38.97 ± 0.01c	38.66 ± 0.56c	38.05 ± 0.01 cd	38.05 ± 0.01 cd	38.55D
D4	37.14 ± 0.02de	37.15 ± 0.03de	37.18 ± 0.02d	37.19 ± 0.01d	37.22 ± 0.01d	37.18E
Mean	39.62A	39.51AB	39.59AB	39.47B	39.51AB	
*Thickness (mm)*						
D0	6.54 ± 0.03kd	6.63 ± 0.01d	6.75 ± 0.02 dc	6.78 ± 0.02 dc	6.82 ± 0.01 dc	6.70D
D1	6.88 ± 0.01c	6.87 ± 0.01c	6.89 ± 0.04c	6.92 ± 0.02bc	6.93 ± 0.05ab	6.90C
D2	6.92 ± 0.01a	6.94 ± 0.02ab	6.94 ± 0.015ab	6.95 ± 0.01ab	6.98 ± 0.01a	6.94D
D3	6.97 ± 0.02ab	6.96 ± 0.02a	6.95 ± 0.03a	6.97 ± 0.02ab	6.95 ± 0.03ab	6.96A
D4	6.94 ± 0.02a	6.95 ± 0.03a	6.92 ± 0.01ab	6.94 ± 0.04ab	6.93 ± 0.01ab	6.93B
Mean	6.85D	6.87C	6.89B	6.91A	6.92A	
*Spread ratio*						
D0	4.54 ± 0.03a	4.53 ± 0.01a	4.55 ± 0.02a	4.55 ± 0.03a	4.52 ± 0.01a	4.54A
D1	4.35 ± 0.02b	4.37 ± 0.01b	4.36 ± 0.04b	4.32 ± 0.01b	4.33 ± 0.04bc	4.34B
D2	4.32 ± 0.01bc	4.33 ± 0.01bc	4.34 ± 0.01bc	4.33 ± 0.01bc	4.36 ± 0.03bc	4.34B
D3	4.61 ± 0.61a	3.96 ± 0.02bc	3.94 ± 0.04c	3.97 ± 0.02 cd	3.95 ± 0.03 cd	4.088C
D4	3.14 ± 0.02de	3.19 ± 0.01de	3.20 ± 0.01e	3.20 ± 0.01e	3.22 ± 0.02e	3.19D
Mean	4.19A	4.07B	4.08B	4.07B	4.08B	

*Note:* Values are Mean and SD (*n* = 3). Different letters in the columns of each parameter indicate significant differences (*p* < 0.05) among treatments according to LSD test.

Abbreviations: D0, Control; D1, 5% PSF; D2, 10% PSF; D3, 15% PSF; D4, 20% PSF; PSF, pumpkin seed flour.

The thicknesses of the PSF biscuits were analyzed under different treatments: D0 (6.54–6.82 mm), D1 (6.88–6.93 mm), D2 (6.92–6.98 mm), D3 (6.97–6.95 mm), and D4 (6.94–6.93 mm) during the storage period. The findings of the present study indicated that the maximum thickness of biscuits (6.98 mm) was observed in treatment D2, followed by treatment D1 (6.93 mm), D3 (6.95 mm), D4 (6.93 mm), and D0 (6.82 mm) on day 60. In contrast, the minimum thickness value was noted for biscuits in treatment D0 (6.54 mm), followed by D1 (6.88 mm), D2 (6.92 mm), D3 (6.97 mm), and D4 (6.94 mm) on day 1. The data from the statistical analysis presented that there are no statistically significant differences between the values for sample D0—on days 30 and 45; for sample D1—on days 1, 15, and 30; for sample D2—on days 15, 30, and 45; and for sample D4—on days 1 and 15. According to the results, the D2 and D3 treatments showed the maximum increase in thickness with 10% and 15% incorporation of PSF. Similar findings were reported by Khan et al. (2019), who reported that adding varying concentrations of pumpkin flour affected the physicochemical properties of the biscuits, possibly affecting their thickness. The optimal level of pumpkin flour incorporation was 15%.

The spread ratio factor of PSF biscuits was analyzed for different treatments during the storage period: D0 (4.54–4.52), D1 (4.35–4.33), D2 (4.32–4.36), D3 (4.61–3.95), and D4 (3.14–3.22). The present study's findings indicate that the maximum spread ratio value of biscuits (4.55) was observed in the treatment D0, followed by D1 (4.32), D2 (4.33), D3 (3.97), and D4 (3.20) on day 45. In contrast, the minimum was noted for biscuits D4 (3.14), followed by D0 (4.54), D1 (4.35), D2 (4.32), and D3 (4.61) on day 1. According to the results, the D3 and D4 treatments showed the maximum decrease in the spread ratio, and the D1 and D2 results were close to the D0 (control) treatment. The D1 (control) biscuits had a higher spread ratio, whereas the PSF replacement biscuits had ratio values close to those of the control. The dough can flow and spread more during baking because WF produces a stronger gluten network and has a balanced starch to protein composition. This is most likely why control biscuits (D1) had a higher spread ratio. This means that adding PSF would result in high‐quality biscuits.

These results are comparable to those reported by Dhiman et al. (2018); the spread ratio of cookies decreased nonsignificantly when pumpkin seed powder was added. The spread ratios for the cookies with 10%, 20%, and 30% pumpkin seed powder were 0.331, 0.328, 0.321, and 0.313, respectively. Because the spread factor is the ratio of the biscuit width to thickness, a drop in the biscuit thickness in the composite flours decreased the spread factor.

Another similar study by Khan et al. (2019) reported that pumpkin pomace powder could be used to produce cookies made of two types of wheat. Cookies produced with Greek‐79 WF and pumpkin pomace powder at 0%, 10%, 15%, 20%, and 25% showed spread ratios of 9.14, 5.98, 5.57, 5.37, and 5.19, respectively. This suggests that adding more pumpkin pomace powder to the recipe significantly reduces the spread ratio of the cookies.

### 3.11 Sensory Analysis of PSF Biscuits

**FIGURE 12:**
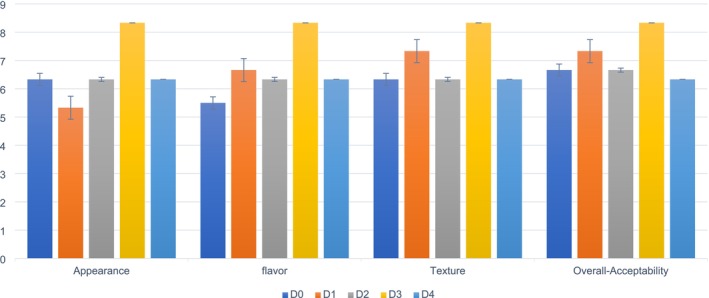
Sensory analysis of PSF biscuits. D0, Control; D1, 5% PSF; D2, 10% PSF; D3, 15% PSF; D4, 20% PSF; PSF, pumpkin seed flour.

The present study's findings indicate that the D3 (8.33–7.33) treatment had the highest appearance score in biscuits, followed by D0 (6.33–6.33), D1 (5.33–6.33), D2 (6.33–6.33), and D4 (6.33–5.33). Meanwhile, the lowest appearance score was marked in treatment D1 (5.33–6.33) biscuits, followed by D0 (6.33–6.33), D2 (6.33–6.33), D3 (8.33–7.33), and D4 (6.33–5.33) from Day 1–60, as shown in Figure 12. The mean appearance values of PSF biscuits differed significantly (*p* ≤ 0.05).

These results were similar to the findings of Khan et al. (2019), who reported that adding 15% PSF to biscuits improved the sensory score of their appearance category after storage. Another similar study by Zamłyńska‐Kowal et al. (2018) concluded that incorporating 15% pumpkin flour improved the sensory appearance of yeast dough biscuits, ranking them at the best quality level during storage.

The findings of the present study indicated that D3 (8.33–7.33) treatment had the highest flavor score in PSF biscuits, followed by D0 (5.50–6.66), D1 (6.66–6.66), D2 (6.33–6.33), and D4 (6.33–5.33). Meanwhile, the lowest flavor score was observed on D0 (5.50–6.66) in PSF biscuits, followed by D1 (6.66–6.66), D2 (6.33–6.33), D3 (8.33–7.33), and D4 (6.33–5.33) from day 0–day 60. The mean flavor values of the PSF biscuits were significantly different (*p* ≤ 0.05). In comparison to D0, D1, D3, and D4, the D3 treatment replaced 15% of the PSF in biscuits and obtained good ratings for flavor according to the panelists' assessment.

According to Kumari et al. (2021), adding 30% PSF to biscuits increases their flavor sensory rating. These results were consistent with their findings. This improvement was ascribed to the nutrient‐rich composition of pumpkin seeds, which offers organoleptic qualities and health advantages. In addition to providing health advantages (such as vital fatty acids, vitamins, and minerals), adding nutrient‐rich PSF to biscuits improves flavor complexity, texture, and aroma, all of which increase the product's customer appeal. In conclusion, the nutrient‐rich composition creates a complete sensory experience by improving organoleptic qualities through biochemical contributions to taste, texture, aroma, and visual appeal. Giami et al. (2005) concluded that biscuits containing 15% PSF retained their flavor sensory scores even after storage, resulting in enhanced nutritional content.

The findings of the present study indicate that the D3 (8.33–7.33) treatment had the highest texture score in PSF biscuits, followed by D0 (6.33–7.00), D1 (7.33–6.66), D2 (6.33–6.33), and D4 (6.33–5.33). The lowest texture score was observed in D4 (6.33–5.33), followed by D0 (6.33–7.00), D1 (7.33–6.66), D2 (6.33–6.33), and D3 (8.33–7.33) biscuits from day 0–60. The mean texture values of the PSF biscuits differed significantly (*p* ≤ 0.05). According to the panelists' grading, the D3 treatment received good, reasonable scores regarding texture in biscuits, with a 15% replacement of PSF compared with D0, D1, D3, and D4 during the storage period.

These results are similar to the findings of Kumari et al. (2021), who mentioned that biscuits containing 15% germinated PSF retain their texture sensory scores during storage, boosting the product's nutritional value and potential health benefits. Hussain et al. (2023) concluded that incorporating 15% PSF into biscuits did not negatively impact texture sensory scores over time, resulting in enhanced quality biscuits with desirable rheological properties.

The present study outcomes indicated that D3 (8.33–7.33) treatment had the highest overall acceptability score for PSF biscuits, followed by D0 (6.66–7.66), D1 (7.33–7.00), D2 (6.66–6.33), and D4 (6.33–5.33). Meanwhile, the lowest overall acceptability score was observed in D4 (6.33–5.33) biscuits, followed by D0 (6.66–7.66), D1 (7.33–7.00), D2 (6.66–6.33), and D3 (8.33–7.33) from days 0–60. The mean values of the overall acceptability of the PSF biscuits were significantly different (*p* ≤ 0.05). In comparison to D0, D1, D3, and D4, the D3 treatment obtained excellent ratings for general acceptability in biscuits during storage with 15% replacement of PSF, based on panelists' grading.

## 4 Conclusion

In conclusion, the addition of PSF to WF significantly alters dough mixing behaviorand can be effectively used to produce high‐quality biscuits. Among the five treatments, the D3 formulation containing 15% PSF demonstrated superior nutritional, textural, physical, color, and sensorial properties during storage. The inclusion of PSF enhanced the nutritional and sensorial properties of the biscuits, with the D3 treatment showing the most favorable characteristics. This study has some limitations, such as the relatively short 60‐day storage period, regional variations in ingredient properties that might not account for changes over a longer shelf life, and the possibility of variability in ingredient properties caused by regional variations in the composition of pumpkin seeds. Future studies might examine how different PSF processing techniques affect product quality, the impacts of extended storage times to better understand shelf‐life dynamics, and use larger and more varied panels to gauge consumer acceptability.

## Author Contributions

**Muhammad Tayyab Arshad:** writing – original draft (equal). **Nosiba S. Basher:** conceptualization (equal), data curation (equal). **Ali Ikram:** supervision (equal), writing – review and editing (equal). **Muhammad Ahmad:** methodology (equal). **Nasir A. Ibrahim:** data curation (equal), formal analysis (equal). **Ammar Al‐Farga:** data curation (equal), visualization (equal). **Kodjo Théodore Gnedeka:** validation (equal).

## Ethics Statement

This study was approved by The University of Lahore. The research ethical committee was numbered as REC‐UOL‐/9/111/23.

## Consent

Informed consent was obtained from all panelists of the sensory evaluators in written form. There has been no medical research in which human participants are involved.

## Conflicts of Interest

The authors declare no conflicts of interest.

## Data Availability

Data supporting the findings of this study are available upon request from the corresponding author. The data were not publicly available due to privacy or ethical restrictions.

## References

[fsn371089-bib-0001] Adelerin, R. O. , O. O. Awolu , B. O. T. Ifesan , and M. U. Nwaogu . 2024. “Pumpkin‐Based Cookies Formulated From Optimized Pumpkin Flour Blends: Nutritional and Antidiabetic Potentials.” Food and Humanity 2: 100215.

[fsn371089-bib-0002] Adeola, A. A. , S. O. Ayansina , G. I. Kayode , and A. R. Aderounmu . 2020. “Effect of Storage on Physical, Chemical, Microbial and Sensory Properties of a Formulated Biscuit Prepared From Sweetpotato‐Pigeonpea‐Banana Flour Blend.” Alexandria Journal of Food Science and Technology 17, no. 2: 1–10.

[fsn371089-bib-0003] Aktaş, N. , and K. E. Gerçekaslan . 2024. “Pumpkin ( *Cucurbita pepo* L.) Pulp Flour as a Source of Dietary Fiber: Chemical, Physicochemical and Technological Properties.” Akademik Gıda 22, no. 1: 14–22.

[fsn371089-bib-0004] Alshehry, G. A. 2020. “Preparation and Nutritional Properties of Cookies From the Partial Replacement of Wheat Flour Using Pumpkin Seeds Powder.” World 9, no. 2: 48–56.

[fsn371089-bib-0005] American Association of Cereal Chemists. Approved Methods Committee . 2000. Approved Methods of the American Association of Cereal Chemists. Vol. 1. American Association of Cereal Chemists.

[fsn371089-bib-0007] AOAC , ed. 2012. Official Methods of Analysis. 18th ed. AOAC International.

[fsn371089-bib-0008] Apostol, L. , C. Moșoiu , C. S. Iorga , and S. Á. Martínez . 2020. “Effect of the Addition of Pumpkin Powder on the Physicochemical Qualities and Rheological Properties of Wheat Flour.” Romanian Biotechnological Letters 25, no. 3: 1594–1600.

[fsn371089-bib-0009] Ayo, J. A. , P. Tailem , P. Osabo , and S. Kave . 2024. “Effects of Added Fluted Pumpkin Seed Flour on the Chemical Composition, Physical and Sensory Quality of Acha‐Wheat Bread.” Asia Pacific Journal of Sustainable Agriculture, Food and Energy 12, no. 2: 77–86.

[fsn371089-bib-0010] Aziz, A. , S. Noreen , W. Khalid , et al. 2023. “Pumpkin and Pumpkin Byproducts: Phytochemical Constitutes, Food Application and Health Benefits.” ACS Omega 8, no. 26: 23346–23357. 38170139 10.1021/acsomega.3c02176 PMC10761000

[fsn371089-bib-0011] Balaswamy, K. , P. G. Prabhakara Rao , G. Sulochanamma , A. Nagender , and K. Sathiya Mala . 2022. “Stability of β‐Carotene in Pumpkin Flour Fortified Vermicelli.” Indian Journal of Nutrition and Dietetics 59, no. 3: 310–322.

[fsn371089-bib-0012] Baltacıoğlu, C. , and N. Ülker . 2017. “Investigation of the Effect of Whole Pumpkin ( *Cucurbita pepo* L.) Powder on Quality Criteria of Biscuits.” Turkish Journal of Agriculture ‐ Food Science and Technology 5: 439–1445.

[fsn371089-bib-0070] Baqoyeva, S. S. , B. N. Amanov , and Z. M. Amonova . 2023. “Using Pumpkin Flour in Cookie Production. Multidisciplinary.” Journal of Science and Technology 3, no. 4: 119–125.

[fsn371089-bib-0013] Batool, M. , M. Ranjha , U. Roobab , et al. 2022. “Nutritional Value, Phytochemical Potential, and Therapeutic Benefits of Pumpkin (Cucurbita sp.).” Plants 11, no. 11: 1394. 35684166 10.3390/plants11111394 PMC9182978

[fsn371089-bib-0014] Bayramov, E. , S. Aliyev , A. Gasimova , S. Gurbanova , and I. Kazimova . 2022. “Increasing the Biological Value of Bread Through the Application of Pumpkin Puree.” Eastern‐European Journal of Enterprise Technologies 116, no. 11: 58–68.

[fsn371089-bib-0015] Bhasker, V. 2016. “Development and Evaluation of High Protein and Low Gluten Biscuits.” International Journal of Innovative Technology and Research 4, no. 4: 5329–5335.

[fsn371089-bib-0016] Chantaro, P. , S. Devahastin , and N. Chiewchan . 2008. “Production of Antioxidant High Dietary Fiber Powder From Carrot Peels.” LWT‐Food Science and Technology 41, no. 10: 1987–1994.

[fsn371089-bib-0017] Costa, L. L. , P. H. F. Tomé , F. B. B. Jardim , et al. 2018. “Physicochemical and Rheological Characterization of Pan Bread Made With Pumpkin Seed Flour.” International Food Research Journal 25, no. 4: 1489–1496.

[fsn371089-bib-0018] Das, S. , M. Ghosh , and P. Chakraborty . 2021. “Study of the Utilization of “Pumpkin Seed” for the Production of Nutritionally Enriched Biscuits.” International Journal of Food Science and Nutrition 6, no. 1: 63–67.

[fsn371089-bib-0019] Davoudi, Z. , M. Shahedi , and M. Kadivar . 2020. “Effects of Pumpkin Powder Addition on the Rheological, Sensory, and Quality Attributes of Taftoon Bread.” Cereal Chemistry 97, no. 5: 904–911.

[fsn371089-bib-0020] Dhiman, A. K. , K. Bavita , S. Attri , and P. Ramachandran . 2018. “Preparation of Pumpkin Powder and Pumpkin Seed Kernel Powder for Supplementation in Weaning Mix and Cookies.” International Journal of Chemical Studies 6, no. 5: 167–175.

[fsn371089-bib-0021] Elinge, C. M. , A. Muhammad , F. A. Atiku , et al. 2012. “Proximate, Mineral and Anti‐Nutrient Composition of Pumpkin ( *Cucurbita pepo* L) Seeds Extract.” International Journal of Plant Research 2, no. 5: 146–150.

[fsn371089-bib-0022] Fagan, C. C. , C. D. Everard , and K. McDonnell . 2011. “Prediction of Moisture, Calorific Value, Ash and Carbon Content of Two Dedicated Bioenergy Crops Using Near‐Infrared Spectroscopy.” Bioresource Technology 102, no. 8: 5200–5206. 21349705 10.1016/j.biortech.2011.01.087

[fsn371089-bib-0023] Gao, D. , A. Helikh , Z. Duan , Y. Liu , and F. Shang . 2022. “Development of Pumpkin Seed Meal Biscuits.” Eastern‐European Journal of Enterprise Technologies 2, no. 11: 116.

[fsn371089-bib-0024] Giami, S. Y. , S. C. Achinewhu , and C. Ibaakee . 2005. “The Quality and Sensory Attributes of Cookies Supplemented With Fluted Pumpkin (*Telfairia occidentalis* Hook) Seed Flour.” International Journal of Food Science & Technology 40, no. 6: 613–620.

[fsn371089-bib-0026] Hagos, M. , B. S. Chandravanshi , M. Redi , and E. Yayi . 2023. “Determination of Total Phenolic, Total Flavonoid, Ascorbic Acid Contents and Antioxidant Activity of Pumpkin Flesh, Peel and Seeds From Different Regions of Ethiopia.” Bulletin of the Chemical Society of Ethiopia 37, no. 5: 1093–1108.

[fsn371089-bib-0025] Hagos, M. , E. E. Yaya , B. S. Chandravanshi , and M. Redi‐Abshiro . 2022. “Analysis of Volatile Compounds in Flesh, Peel and Seed Parts of Pumpkin ( *Cucurbita maxima* ) Cultivated in Ethiopia Using Gas Chromatography‐Mass Spectrometry (GC‐MS).” International Journal of Food Properties 25, no. 1: 1498–1512.

[fsn371089-bib-0027] Hussain, A. , T. Kausar , J. Aslam , et al. 2023. “Physical and Rheological Studies of Biscuits Developed With Different Replacement Levels of Pumpkin ( *Cucurbita maxima* ) Peel, Flesh, and Seed Powders.” Journal of Food Quality 2023, no. 1: 4362094.

[fsn371089-bib-0028] Hussain, A. , T. Kausar , A. Din , et al. 2021. “Determination of Total Phenolic, Flavonoid, Carotenoid, and Mineral Contents in Peel, Flesh, and Seeds of Pumpkin ( *Cucurbita maxima* ).” Journal of Food Processing and Preservation 45, no. 6: e15542.

[fsn371089-bib-0029] Ikram, A. , A. Rasheed , A. Ahmad Khan , et al. 2024. “Exploring the Health Benefits and Utility of Carrots and Carrot Pomace: A Systematic Review.” International Journal of Food Properties 27, no. 1: 180–193.

[fsn371089-bib-0030] Ikram, A. , F. Saeed , M. Afzaal , et al. 2021. “Nutritional and End‐Use Perspectives of Sprouted Grains: A Comprehensive Review.” Food Science & Nutrition 9, no. 8: 4617–4628. 34401108 10.1002/fsn3.2408 PMC8358358

[fsn371089-bib-0031] Jayalath, K. A. T. K. , E. Mendis , and A. A. D. Madushan . 2024. “Evaluation of Physical and Sensory Properties of Biscuits Made From Blended Wheat Flour and Pumpkin Powder.” Tropical Agricultural Research 35, no. 4: 288–295.

[fsn371089-bib-0032] Jukić, M. , J. Lukinac , J. Čuljak , M. Pavlović , D. Šubarić , and D. Koceva Komlenić . 2019. “Quality Evaluation of Biscuits Produced From Composite Blends of Pumpkin Seed Oil Press Cake and Wheat Flour.” International Journal of Food Science & Technology 54, no. 3: 602–609.

[fsn371089-bib-0033] Kaur, M. , K. S. Sandhu , A. Arora , and A. Sharma . 2015. “Gluten‐Free Biscuits Prepared From Buckwheat Flour Incorporating Various Gums: Physicochemical and Sensory Properties.” LWT‐ Food Science and Technology 62, no. 1: 628–632.

[fsn371089-bib-0034] Khan, M. A. , C. Mahesh , P. Vineeta , G. K. Sharma , and A. D. Semwal . 2019. “Effect of Pumpkin Flour on the Rheological Characteristics of Wheat Flour and on Biscuit Quality.” Journal of Food Processing & Technology 10, no. 9: 1–6.

[fsn371089-bib-0035] Kulkarni, A. S. , and D. C. Joshi . 2013. “Effect of Replacement of Wheat Flour With Pumpkin Powder on Textural and Sensory Qualities of Biscuit.” International Food Research Journal 20, no. 2: 587.

[fsn371089-bib-0036] Kumari, N. , S. C. Sindhu , V. Kumari , and V. Rani . 2020. “Nutritional Evaluation of Developed Value Added Biscuits Incorporating Germinated Pumpkin Seed Flour.” Journal of Pharmacognosy and Phytochemistry 9, no. 5: 2802–2806.

[fsn371089-bib-0037] Kumari, N. , S. C. Sindhu , V. Rani , and V. Kumari . 2021. “Shelf Life Evaluation of Biscuits and Cookies Incorporating Germinated Pumpkin Seed Flour.” International Journal of Current Microbiology and Applied Sciences 10, no. 1: 1436–1443.

[fsn371089-bib-0038] Kundu, H. , R. B. Grewal , A. Goyal , N. Upadhyay , and S. Prakash . 2014. “Effect of Incorporation of Pumpkin (*Cucurbita moshchata*) Powder and Guar Gum on the Rheological Properties of Wheat Flour.” Journal of Food Science and Technology 51: 2600–2607. 25328201 10.1007/s13197-012-0777-x PMC4190245

[fsn371089-bib-0041] Malkanthi, H. H. A. , and S. H. Umadevi . 2018. “Effect of Dried Pumpkin Pulp and Seed Powder on Physical, Chemical and Sensory Properties of Biscuits.” International Journal of Science and Research 7, no. 8: 32–34.

[fsn371089-bib-0042] Mazaal, A. M. , W. F. Abas , and M. A. Mousa . 2022. “Preparation and Evaluation of Healthy Biscuits Using Germinated Wheat Flour and Germinated PSF.” Neuroquantology 20, no. 4: 244.

[fsn371089-bib-0043] Meilgaard, M. , G. Vance Civille , and T. Carr . 2007. Sensory Evaluation Techniques. 4th ed. Pp. 7–8, 173–179. CRC Press.

[fsn371089-bib-0044] Melese, A. D. , and E. O. Keyata . 2023. “Physiochemical Properties and Sensory Acceptability of Bread Developed From Wheat‐Pumpkin and Common Bean Flour.” Journal Of Food Chemistry And Nanotechnology 9, no. 1: 13–20.

[fsn371089-bib-0045] Minarovičová, L. , M. Lauková , Z. Kohajdová , J. Karovičová , and V. Kuchtová . 2017. “Effect of Pumpkin Powder Incorporation on Cooking and Sensory Parameters of Pasta.” Potravinarstvo Slovak Journal of Food Sciences 11, no. 1: 373–379.

[fsn371089-bib-0046] Mohamed, G. F. , A. M. Sulieman , N. G. Soliman , and S. S. Bassiuny . 2014. “Fortification of Biscuits With Fish Protein Concentrate.” World Journal of Dairy & Food Sciences 9, no. 2: 242–249.

[fsn371089-bib-0047] Moraes, M. S. , A. J. M. Queiroz , R. M. F. Figueirêdo , et al. 2024. “New Cookie Formulations Using Germinated Pumpkin Seed Flour–Increase Its Nutritional and Sensory Value.” International Food Research Journal 31, no. 4: 835–846.

[fsn371089-bib-0048] Nyam, K. L. , M. Lau , and C. P. Tan . 2013. “Fibre From Pumpkin ( *Cucurbita pepo* L.) Seeds and Rinds: Physico‐Chemical Properties, Antioxidant Capacity and Application as Bakery Product Ingredients.” Malaysian Journal of Nutrition 19, no. 1: 99–109. 24800388

[fsn371089-bib-0049] Piga, A. , P. Catzeddu , S. Farris , T. Roggio , A. Sanguinetti , and E. Scano . 2005. “Texture Evolution of “Amaretti” Cookies During Storage.” European Food Research and Technology 221, no. 3: 387–391.

[fsn371089-bib-0051] Rosell, C. M. , E. Santos , and C. Collar . 2009. “Physico‐Chemical Properties of Commercial Fibres From Different Sources: A Comparative Approach.” Food Research International 42, no. 1: 176–184.

[fsn371089-bib-0052] Saeed, F. , M. Afzaal , A. Ikram , et al. 2021. “Exploring the Amino Acid Composition and Vitamin‐B Profile of Buckwheat Varieties.” Journal of Food Processing 45: e15743.

[fsn371089-bib-0053] Saeed, S. M. G. , S. A. Ali , R. Ali , et al. 2020. “Utilization of *Vigna mungo* Flour as Fat Mimetic in Biscuits: Its Impact on Antioxidant Profile, Polyphenolic Content, Storage Stability, and Quality Attributes.” Legume Science 2, no. 4: e58.

[fsn371089-bib-0054] Sangnark, A. , and A. Noomhorm . 2003. “Effect of Particle Sizes on Functional Properties of Dietary Fibre Prepared From Sugarcane Bagasse.” Food Chemistry 80, no. 2: 221–229.

[fsn371089-bib-0055] Senarathna, H. W. U. N. , and S. B. Navaratne . 2017. “Determination of the Organoleptic Quality of Hard Dough Biscuits During the Shelf Life by Chemical Analysis.” International Journal of Advanced Engineering Research and Science 4, no. 1: 221–230.

[fsn371089-bib-0056] Sengul, M. , H. Yildiz , N. Gungor , B. Cetin , Z. Eser , and S. Ercisli . 2009. “Total Phenolic Content, Antioxidant and Antimicrobial Activities of Some Medicinal Plants.” Pakistan Journal of Pharmaceutical Sciences 22, no. 1: 102–106. 19168430

[fsn371089-bib-0057] Sharma, P. , G. Kaur , B. A. Kehinde , N. Chhikara , A. Panghal , and H. Kaur . 2020. “Pharmacological and Biomedical Uses of Extracts of Pumpkin and Its Relatives and Applications in the Food Industry: A Review.” International Journal of Vegetable Science 26, no. 1: 79–95.

[fsn371089-bib-0058] Shevchenko, A. , V. Drobot , and O. Galenko . 2022. “Use of PSFin Preparation of Bakery Products.” Ukrainian Food Journal 11, no. 1: 90–101.

[fsn371089-bib-0059] Shukla, R. N. , A. A. Mishra , and A. K. Gautam . 2016. “Development of Protein Enriched Biscuit Fortified With Green Gram Flour.” Food Science Research Journal 7, no. 1: 112–118.

[fsn371089-bib-0060] Singh, A. , and V. Kumar . 2022. “Nutritional, Phytochemical, and Antimicrobial Attributes of Seeds and Kernels of Different Pumpkin Cultivars.” Food Frontiers 3, no. 1: 182–193.

[fsn371089-bib-0061] Sowbhagya, H. B. , P. F. Suma , S. Mahadevamma , and R. N. Tharanathan . 2007. “Spent Residue From Cumin–A Potential Source of Dietary Fiber.” Food Chemistry 104, no. 3: 1220–1225.

[fsn371089-bib-0063] Suleman, D. , S. Bashir , F. U. Hassan Shah , et al. 2023. “Nutritional and Functional Properties of Cookies Enriched With Defatted Peanut Cake Flour.” Cogent Food & Agriculture 9, no. 1: 2238408.

[fsn371089-bib-0071] Tomsone, L. , Z. Kruma , and R. Galoburda . 2012. “Comparison of Different Solvents and Extraction Methods for Isolation of Phenolic Compounds from Horseradish Roots (Armoracia rusticana).” World Academy of Science, Engineering and Technology 64, no. 4: 903–908.

[fsn371089-bib-0064] Tufail, T. , F. Saeed , M. U. Arshad , et al. 2020. “Exploring the Effect of Cereal Bran Cell Wall on Rheological Properties of Wheat Flour.” Journal of Food Processing and Preservation 44, no. 3: e14345.

[fsn371089-bib-0065] Van Toan, N. , and V. Q. Anh . 2018. “Preparation and Improved Quality Production of Flour and the Made Biscuits From Purple Sweet Potato.” Journal of Food and Nutrition 4: 1–14.

[fsn371089-bib-0066] Wal, A. , M. R. Singh , A. Gupta , S. Rathore , R. R. Rout , and P. Wal . 2024. “Pumpkin Seeds (Cucurbita spp.) as a Nutraceutical Used in Various Lifestyle Disorders.” Natural Products Journal 14, no. 1: 118–137.

[fsn371089-bib-0069] Wang, S. , Y. Fang , Y. Xu , et al. 2022. “The Effects of Different Extraction Methods on Physicochemical, Functional and Physiological Properties of Soluble and Insoluble Dietary Fiber from Rubus chingiiHu. Fruits.” Journal of Functional Foods 93: 105081. 10.1016/j.jff.2022.105081.

[fsn371089-bib-0067] Yetesha, A. , B. S. Chandravanshi , and W. Yohannes . 2023. “Major and Heavy Metals Contents and Health Risk Assessment of Pumpkin Peel, Flesh and Seed by Microwave Plasma‐Atomic Emission Spectroscopy.” Bulletin of the Chemical Society of Ethiopia 37, no. 3: 533–551.

[fsn371089-bib-0068] Zamłyńska‐Kowal, A. , E. Kusińska , E. Sosińska‐Leszczyńska , and M. Panasiewicz . 2018. “Influence of Pumpkin Flour Addition on Textural Properties of Yeast Dough.” Acta Agrophysica 25, no. 3: 317–327.

